# Waste-derived zeolite from sugarcane bagasse ash and water treatment plant sludge for sustainable industrial dye removal

**DOI:** 10.1007/s11356-026-37455-y

**Published:** 2026-02-04

**Authors:** Joana Eliza de Santana, Antônio Elias dos Santos Neto, Fábio Gabriel Silva de Andrade, Aldebarã Fausto Ferreira, Marcos Gomes Ghislandi, Maurício Alves da Motta Sobrinho

**Affiliations:** 1 Chemical Engineering Department, Federal University of Pernambuco, Recife, PE 50740-590 Brazil; 2 Department of Fundamental Chemistry, Federal University of Pernambuco, Recife, PE 50740-590 Brazil; 3 Engineering Campus, Federal Rural University of Pernambuco, Cabo de Santo Agostinho, PE 54518-430 Brazil

**Keywords:** Industrial waste valorization, Dye removal, Decontamination, Circular economy, Sustainable adsorbents, Acid Red 27

## Abstract

**Abstract:**

This study presents a sustainable route for synthesizing zeolites by valorizing two abundant industrial residues: sugarcane bagasse ash and water treatment plant sludge. The synthesized material, primarily composed of sodalite as confirmed by XRD and SEM, was applied for the removal of Acid Red 27 (AR27), a synthetic dye widely used in the food, cosmetics, and household product industries. The adsorption kinetics followed a pseudo-second-order model, suggesting dependence on active site availability, while isotherm analysis indicated multilayer adsorption consistent with the BET model, with an adsorption capacity reaching 250 mg·g^−1^. Experiments conducted with competitive anions suggest the adsorption mechanism in the first layer predominantly involved electrostatic interactions; the dye structure suggests π–π stacking for subsequent layers. Coexisting anions, particularly sulfate and bicarbonate, significantly hindered AR27 uptake due to competitive adsorption. Importantly, the adsorbent maintained its performance over three regeneration cycles using a diluted NaOH solution (0.01 M). Compared to granular and powdered activated carbons, the synthesized zeolite exhibited superior performance, especially at medium to high contaminant loads. These findings highlight the potential of waste-derived zeolites as a low-cost, efficient, and environmentally friendly material for wastewater treatment, contributing to circular economy strategies and sustainable resource management.

**Graphical Abstract:**

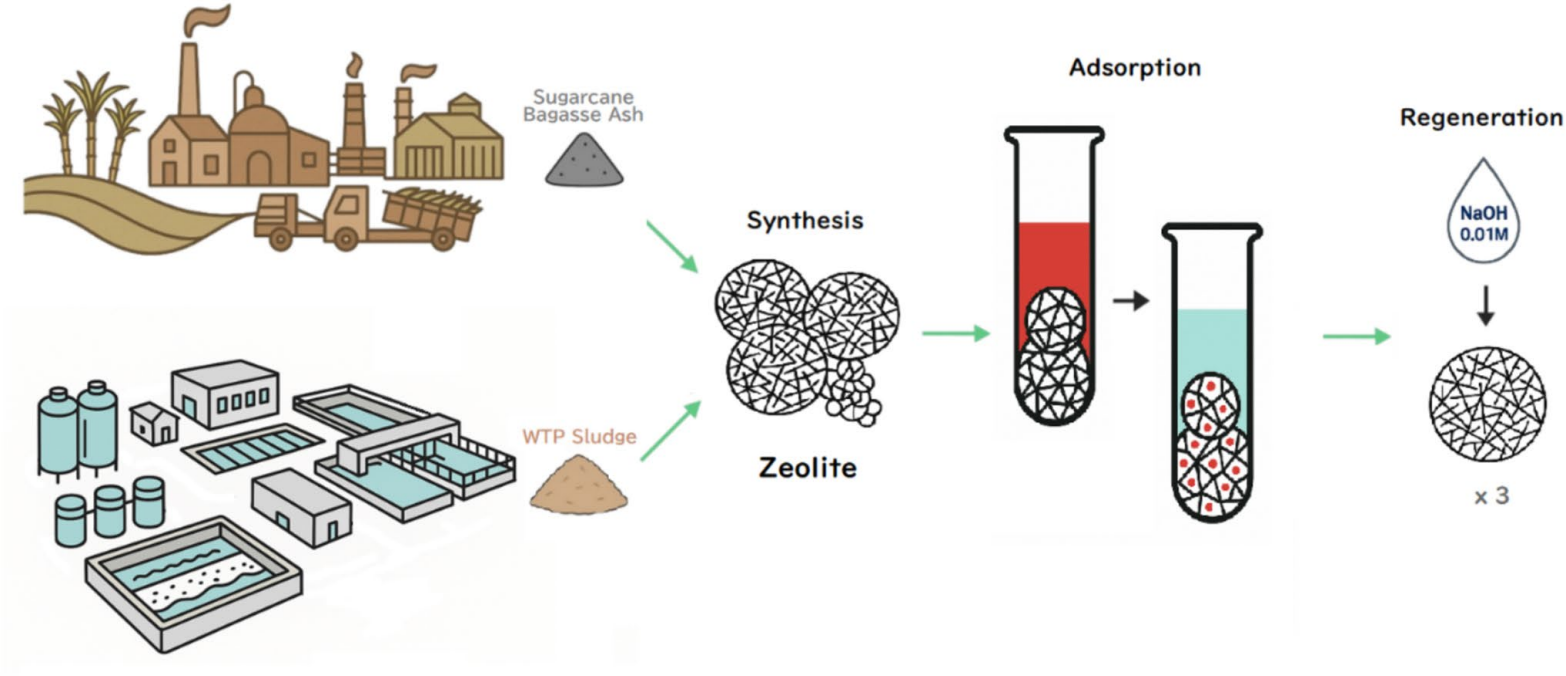

**Supplementary information:**

The online version contains supplementary material available at 10.1007/s11356-026-37455-y.

## Introduction

Zeolites are microporous crystalline materials composed of interconnected SiO_4_ and AlO_4_ tetrahedra (Grecco et al. 2013), and they are highly valued for their specific properties such as morphology and surface structure (Jha and Singh 2016; Rahman and Hayat 2019). Due to these characteristics, they are widely employed as catalysts, ion exchangers, and, notably, as adsorbents for environmental applications like wastewater treatment (Davis 2013). In response to increasing environmental concerns and the demand for sustainable production routes, the synthesis of zeolites from industrial residues has gained growing attention. This approach simultaneously reduces the environmental burden of conventional synthesis processes and promotes the valorization of wastes that would otherwise be discarded.

Among potential raw materials, water treatment plant sludge (WTPS) and sugarcane bagasse ash (SCBA) stand out due to their complementary chemical compositions for zeolite obtention. WTPS is generated during coagulation and flocculation processes and typically accumulates in sedimentation units, posing serious disposal challenges, especially in urban areas (Baydum et al. 2021). In Brazil alone, more than 78 million tons (dry basis) of WTPS were generated in 2015 (Brazil 2022). When aluminum-based coagulants are used, the sludge becomes enriched in aluminum, a key precursor for zeolite formation (Di Bernardo et al. 2012). Despite various reuse attempts, such as soil conditioning, cement and brick production, pavement materials, and coagulant recovery (Ahmadi et al. 2023; Mattoso et al. 2024; Arisiketty and Vijayarengan 2024; Takao et al. 2024), most of this waste remains inadequately managed; only 2.38% of the amount produced in 2008 was reused, while 67.88% was improperly disposed of in water resources (IBGE 2010). Given population growth and increased demand for water supply, it is imperative to adopt sustainable and highly scalable management models.

SCBA represents a parallel environmental challenge. Brazil, the world’s largest sugarcane producer (FAOSTAT 2025), with an estimated 676.9 million tons harvested in 2024/2025 (CONAB 2025) , generates substantial amounts of bagasse, which corresponds to approximately 30% of cane mass (Sriatun et al. 2018). The combustion of bagasse in high-pressure boilers produces an ash rich in silica, making SCBA a suitable silicon source for zeolite synthesis (Moisés et al. 2013). Each ton of burned bagasse yields 25–40 kg of ash (Xu et al. 2018), resulting in millions of tons of SCBA annually. Improper disposal of this residue can cause significant environmental impacts, reinforcing the need for valorization strategies.

Together, WTPS and SCBA constitute accessible and complementary sources of aluminum and silicon for zeolite synthesis. Their combined use not only mitigates environmental liabilities but also supports circular economy principles by reducing dependence on virgin raw materials (Geissdoerfer et al. 2017). Although several studies have reported zeolite synthesis from individual industrial wastes (Purnomo et al. 2012; Collins et al. 2020; Praipipat et al. 2023; Nuhu et al. 2024), synergistic approaches combining residues with complementary compositions remain scarcely explored. No study has been identified that integrates silica-rich SCBA and aluminum-rich WTPS for the synthesis of zeolites aimed at environmental remediation. Addressing this gap offers opportunities to intensify waste valorization and advance more efficient circular strategies.

Unlike conventional approaches that rely on a single residue supplemented with commercial reagents, this study proposes a novel dual-waste route for zeolite synthesis using SCBA and WTPS as low- or zero-cost precursors of silicon and aluminum. This strategy contrasts with the high cost of commercial reagents (Hussain et al. 2021; Shin and Kim 2024) and aligns with green chemistry principles by minimizing the use of pristine chemical inputs.

In parallel, the release of industrial dyes into aquatic environments remains a major environmental concern due to their persistence, toxicity, and potential for bioaccumulation and biomagnification (Pinedo-Hernández et al. 2012; Ebrahimpoor et al. 2019; Şenol et al. 2024). Conventional treatment technologies (such as electrochemical, oxidative, membrane, and biological processes) often involve high costs, operational complexity, or the generation of secondary pollutants (Salman-Naeem et al. 2018; Satyam and Patra 2025). Adsorption emerges as an attractive alternative due to its simplicity, efficiency, and minimal by-product formation (Sahoo and Prelot 2020).

The efficiency of adsorption processes strongly depends on the adsorbent material. Zeolites stand out among conventional adsorbents, such as activated carbon and graphene (Araujo et al. 2020) and biopolymer composites (Şenol et al. 2023; Araujo et al. 2024), due to their high thermal and chemical stability, regenerability, and selective adsorption behavior (Pérez-Botella et al. 2022).

From a regulatory perspective, industrial effluent discharges are governed by environmental agencies, including CONAMA Resolution No. 430/2011 in Brazil (Brazil 2011), which establishes limits for parameters such as color, toxicity, and Chemical Oxygen Demand (COD). Although no specific discharge limits exist for individual dyes such as Acid Red 27 (AR27), its classification as an emerging contaminant underscores increasing environmental concern. The removal of AR27 directly contributes to reductions in color, COD, and toxicity, which are central to regulatory compliance.

In this context, the present study proposes the valorization of WTPS and SCBA for the synthesis of zeolites specifically designed for dye adsorption. Acid Red 27, widely used in food additives, cleaning products, cosmetics, and textiles (Chhabra et al. 2015), was selected as a model pollutant. This integrated strategy aims to provide a low-cost and sustainable solution for wastewater remediation through the development of efficient waste-derived adsorbents.

The findings are expected to support the development of scalable treatment processes and to mitigate the environmental impacts associated with the production and use of commercial chemical reagents, enabling a sustainable cycle for the synthesis and application of zeolites in pilot- and industrial-scale systems, either as a standalone unit or as a polishing step. Moreover, the proposed dual-residue approach can be extended to the removal of other emerging contaminants, such as heavy metals and pharmaceuticals, reinforcing sustainable material lifecycle practices.

## Experimental

This study employed the following analytical-grade (P.A.) reagents: hydrochloric acid (37%, Química Moderna, Brazil), sodium hydroxide (> 98.0%, Dinâmica, Brazil), sodium chloride (> 99.5%, Fmaia, Brazil), anhydrous sodium sulfate (> 99.0%, Vetec, Brazil), sodium bicarbonate (ACS grade, > 99.7%, Química Moderna, Brazil), aluminum sulfate (> 98.0%, Dinâmica, Brazil), sodium nitrate (> 99.0%, Dinâmica, Brazil), ethanol (99.5%, Neon, Brazil), granular activated carbon (Dinâmica, Brazil), powdered activated carbon (Dinâmica, Brazil), and the synthetic dye Acid Red 27 (Polycrom, Brazil). All reagents were used as received, without undergoing any additional purification steps.

### Sugarcane bagasse ash and WTP sludge

The sugarcane bagasse ash (SCBA) used in this research was supplied by a sugar mill located in Lagoa de Itaenga, Pernambuco, Brazil. In the mill’s industrial process, the bagasse is incinerated in high-pressure boilers operating at approximately 380 °C. The resulting ash is transported via a water flow system to a sedimentation pond. After the decantation stage, the material was drained, oven-dried at 105 °C for 24 h, and subsequently sieved. The portion with particles smaller than 0.25 mm was selected for further use.

The water treatment plant sludge (WTPS) was collected in a dehydrated state from bags stored at the Gurjaú WTP, operated by Companhia Pernambucana de Saneamento (COMPESA), in Cabo de Santo Agostinho, PE, Brazil. The sludge was oven-dried at 105 °C for 24 h, then ground and sieved. As with the SCBA, only the particle fraction smaller than 0.25 mm was utilized, ensuring consistency in material granulometry for the subsequent stages of the study.

### Extraction of silicate and aluminate from SCBA and WTP sludge

SCBA and WTPS were used for the extraction of silicate and aluminate through the alkaline fusion method followed by dissolution (Purnomo et al. 2012). Initially, both materials were calcined at 600 °C for 4 h to eliminate the organic matter present.

For SCBA, 10 g of the fine fraction were mixed with sodium hydroxide (NaOH) at a mass ratio of 1:1.2 (SCBA:NaOH). The mixture was heated at 500 °C for 1 h, resulting in a fused mass that, after cooling to room temperature, was ground into a uniform powder. This fused powder was then mixed with distilled water at a mass ratio of 1:5 (fused mass:water) and subjected to an aging process for 2 h at room temperature under constant stirring on a shaker table at 250 rpm. Subsequently, the suspension was filtered using qualitative filter paper (80 g/m^2^, particle retention 4–12 µm) to separate the solid residues and obtain a clear supernatant. The same procedure was applied to pre-sieved WTP sludge.

The concentrations of silicon, aluminum, sodium, and other metals in the silicate- and aluminate-enriched supernatants were determined according to the Standard Methods for the Examination of Water and Wastewater (SMEWW), 23rd edition, by the American Public Health Association (APHA 2023).

### Zeolite synthesis

Zeolite synthesis was carried out following a methodology adapted from Moisés et al. (2013) and Rozhkovskaya et al. (2021), using the hydrothermal treatment method. Initially, a portion of the silicate-rich supernatant was diluted with distilled water. Then, under vigorous stirring, this diluted supernatant was gradually combined dropwise with the sodium aluminate supernatant. The proportions of each component were adjusted based on the silicon and aluminum content analysis, ensuring that the reaction mixture had a molar Si/Al ratio around 2.0.

After 60 min of stirring at 40 rpm using a magnetic stirrer, the mixture underwent hydrothermal treatment in a stainless-steel reactor internally lined with polytetrafluoroethylene (PTFE), maintained at 100 °C for 2 h. At the end of this period, the autoclaves were cooled, and the resulting material was filtered and washed with distilled water until the pH of the wash water was below 8.0. Subsequently, an additional washing step with analytical-grade ethanol (99.5%) was performed. The resulting solid material was dried in an oven at 60 °C for 24 h and then stored for further analysis. Aluminum sulfate was used as an additional aluminum source, as the WTP sludge did not contain sufficient aluminum to meet the required Si/Al molar ratio around 2.0.

### Characterization of the materials

The physicochemical characterization of sugarcane bagasse ash (SCBA), water treatment plant (WTP) sludge, and zeolite was carried out using a range of analytical techniques. The elemental composition of the materials was analyzed through X-ray fluorescence (XRF) with a handheld XRF device (Bruker S1 Titan 800 model). Morphological features were examined by scanning electron microscopy (SEM) using a VEGA3 (Tescan, CZ) equipment, operated with accelerating voltages between 5 and 20 kV. Samples were mounted on small metallic holders (stubs) using double-sided carbon tape and subsequently coated with a thin gold layer using a Denton Vacuum Desk V (US) sputter coater. Thermogravimetric analyses (TGA) were conducted on a TGA Q50 V6.7 Build 203 instrument, under airflow, across a temperature range from 27 to 1000 °C, at a constant heating rate of 10 °C/min. Nitrogen adsorption–desorption isotherms were collected at 77 K up to relative pressures of 0.99 using a NOVA STATION A system (Quantachrome Instruments). Specific surface area (SBET) was determined using the Brunauer–Emmett–Teller (BET) model, while total pore volume (VT) and average pore diameter were obtained from the adsorption isotherm at a relative pressure of *P*/*Po* = 0.99. X-ray diffraction (XRD) analysis was performed using a Bruker D8 Advance equipment, with Cu-Kα radiation (*λ* = 0.1546 nm), scanning the 2*θ* from 5 to 80°, using a step size of 0.02°/s, with voltage set to 40 kV and current set to 40 mA. The diffraction patterns were evaluated both qualitatively and quantitatively with the HighScore Plus 3.0c software, utilizing the Crystallography Open Database (COD_OCT 2014) as reference. Fourier-transform infrared spectroscopy (FTIR) was conducted in the 400–4000 cm^−1^ range using a Shimadzu IR PRESTIGE-21 spectrometer in transmittance mode; pellet samples were prepared by mixing with dry KBr. Finally, the point of zero charge (pHpzc) of the zeolite was determined based on a modified procedure from Mahmood et al. (2011), using 0.1 M NaCl solution and adjusting the initial pH (pH0) with 0.1 M HCl and 0.1 M NaOH solutions. Measurements were taken using an Instrutherm PH-1500 pH meter.

FTIR and SEM coupled with energy-dispersive X-ray spectroscopy (EDS) analyses were also performed on the material after the adsorption of the AR27 dye, following the same procedures applied to the pristine material, with the purpose of confirming dye retention. The sample obtained from the isotherm experiment was first separated from the solution by centrifugation and then dried in an oven at 60 °C for 24 h prior to analysis.

### Adsorbate

The synthetic dye Acid Red 27 (AR27) [CAS 915-67-3, C.I. 16185; chemical formula: C_20_H_11_N_2_Na_3_O_10_S_3_; molecular weight: 604.5 g·mol^−1^; pKa: 6.5; *λ*ₘₐₓ: 520 nm] was selected for this study. A known mass of the dye was accurately measured using a Quimis Q500B210C analytical balance and dissolved in distilled water to obtain a 1000 mg·L^−1^ stock solution. Working solutions at various concentrations were subsequently prepared by dilution of the stock with distilled water.

The quantification of the working solutions containing the AR27 dye was carried out by molecular absorption spectrophotometry in the ultraviolet/visible (UV/Vis) range, using the Thermo Fisher Scientific Genesys 10-S equipment, at a wavelength of 520 nm.

### Adsorption experiments

To investigate the adsorption of AR27 dye, batch experiments were carried out in a shaker (Marconi MA-420) at 25 ± 1 °C and operated at 300 rpm. Upon completion of each run, the samples were centrifuged to separate the solid material, and the residual dye concentrations were subsequently determined.

For the kinetic studies, different contact times (up to 120 min) were evaluated using dye solutions at various initial concentrations (30, 90, 500, and 1000 mg·L^−1^), covering a representative range for adsorption tests. The experiments were conducted with an adsorbent dosage of 4 g·L^−1^, stirring speed of 300 rpm, temperature of 25 ± 1 °C, and initial pH (pH_0_) of 2. The kinetic models applied included Pseudo-First-Order (PFO), Pseudo-Second-Order (PSO), Pseudo-n-Order (PnO), and Weber–Morris intraparticle diffusion model, with their corresponding equations presented in Table S1 (Section S1 of the supplementary material).

The influence of key parameters on the adsorption process was also evaluated. The influence of the starting pH (pH_0_) on dye removal was investigated across a pH interval from 2 to 10, with pH_0_ modified using 0.1 M HCl or 0.1 M NaOH solutions. The impact of adsorbent dosage on dye removal efficiency and adsorption capacity was assessed by varying the dosage from 1.0 to 8.0 g·L^−1^, keeping all other conditions constant, with each test conducted using a dye solution at a fixed concentration of 30 mg·L^−1^ until adsorption equilibrium was attained.

Equilibrium adsorption experiments were performed with initial dye concentrations ranging up to 1000 mg·L^−1^. Samples were stirred on a shaker until adsorption equilibrium was attained. The resulting data were interpreted using the Langmuir, Freundlich, Dubinin-Radushkevich (D–R), and BET isotherm models through a non-linear fitting approach. The equations for these models are provided in Table S2, located in Section S1 of the supplementary material. Non-linear regression of the adsorption data was conducted using OriginPro© software, adopting a convergence criterion based on a chi-square tolerance of 1.0 × 10^−9^, which corresponds to the difference in reduced chi-square values between two successive iterations.

Furthermore, the influence of coexisting anions (NO_3_^−^, Cl^−^, HCO_3_^−^, and SO_4_^2−^) on the adsorption of AR27 onto the zeolite was assessed. The respective salts (NaNO_3_, NaCl, NaHCO_3_, and Na_2_SO_4_) were introduced into the dye solutions—both individually and in combination—at concentrations of 30 and 90 mg·L^−1^, and the systems were allowed to proceed until adsorption equilibrium was achieved.

For assessing the regeneration and subsequent reuse of the adsorbent, the zeolite was introduced into the dye solution and stirred under controlled conditions until equilibrium was reached. Basic solutions are often applied for the removal of anionic dyes from adsorbents (Patel 2022); therefore, after separating the adsorbent, it was eluted with distilled water and NaOH solutions at different concentrations (0.001 M, 0.01 M, and 0.1 M), with the eluent volume corresponding to 25% of the volume of dye used in the adsorption. The parameters pH, dosage, rotation, and temperature were kept constant, following the conditions of the previous tests. The initial dye concentration was 90 mg·L^−1^. The regeneration process was carried out for three cycles, and the adsorption performance of the regenerated adsorbent was evaluated after each cycle. Each experiment was conducted in duplicate.

For comparative purposes, adsorption equilibrium experiments were also conducted with two widely used commercial adsorbents: granular activated carbon (GAC) and powdered activated carbon (PAC). The experimental conditions for each adsorbent were selected to correspond to their respective optimal adsorption performance, enabling a direct comparison with the synthesized zeolite: 1.5 g·L^−1^ for the zeolitic compound, 2.8 g·L^−1^ for GAC, and 2.5 g·L^−1^ for PAC. All tests were performed with initial AR27 dye concentrations varying between 5 and 1000 mg·L^−1^ at pH_0_ 2, under agitation at 300 rpm and a temperature of 25 ± 1 °C, until adsorption equilibrium was reached. The adsorption data for each adsorbent were fitted to the Langmuir and Freundlich isotherm models using the same non-linear regression procedure described above, allowing a consistent comparison of model parameters and adsorption capacities. For raw sugarcane bagasse ash (SCBA), adsorption data were obtained from de Santana et al. (2024), where experiments were conducted with 4.0 g·L^−1^ of SCBA in AR27 dye solutions at pH_0_ 2, under agitation at 300 rpm and a temperature of 30 ± 1 °C.

The amount of dye adsorbed onto the adsorbent at a given time *q*_*t*_ (mg g^−1^) and at equilibrium *q*_*e*_ (mg g^−1^) were determined using Eqs. (1) and (2), respectively. The removal efficiency (%) of dye was calculated using Eq. (3).

1 $${q}_{t}= \frac{\left({C}_{0}- {C}_{t}\right)\cdot V}{m}$$

 

2 $${q}_{e}= \frac{\left({C}_{0}- {C}_{e}\right)\cdot V}{m}$$

 

3 $$Removal\mathit\;efficiency\mathit=\frac{\mathit{\left({C_0-C_e}\right)}\mathit.\mathit{100}}{{\mathit C}_{\mathit0}}$$

In these equations, *q*_*t*_ and *q*_*e*_ represent the quantity of dye adsorbed (mg·g^−1^) at time t and equilibrium, respectively, *C*_0_ (mg·L^−1^) is the initial dye concentration, *C*_*t*_ (mg·L^−1^) is the dye concentration at time *t*, *C*_*e*_ (mg·L^−1^) corresponds to the dye concentration at equilibrium, *V* represents the volume of the dye solution (L), and *m* is the mass of the adsorbent used (g).

## Results and discussion

### Material characterization

The precursor and synthesized materials were characterized by X-ray fluorescence (XRF), Fourier Transform Infrared Spectroscopy (FTIR), X-ray diffraction (XRD), scanning electron microscopy (SEM), Thermogravimetric Analysis (TGA), textural characterization through nitrogen adsorption isotherms, and determination of the pH at the point of zero charge.

In addition to the main samples discussed in this study, further characterizations were carried out on ashes from different harvests of the same sugarcane mill, ashes from other mills, and sludge from another water treatment plant (WTP), all located in the state of Pernambuco, Brazil. These analyses were aimed at demonstrating that these materials contain sources of silicon and aluminum that allow the zeolite synthesis, thereby supporting their potential as alternative feedstocks. The results of these complementary analyses are presented in Section S2 of the supplementary material.

#### X-ray fluorescence

XRF analysis was used to determine the chemical composition of the SCBA, WTPS, and the zeolitic compound samples. This method is widely employed due to its ability to provide accurate data on the elemental constituents of solid materials.

The results reveal that the precursor samples (ash and sludge) are predominantly composed of silicon, followed by aluminum and iron. Variations in the composition of the ash are influenced by the chemical characteristics of the soil, the variety of sugarcane cultivated, the type of fertilizer applied, the conditions of the bagasse combustion process, and the temperature used (Khalil et al. 2021); while the differences observed in the sludge reflect the quality of the raw water and the chemicals used in the treatment processes (Richter 2001). These factors directly influence the chemical characteristics of the raw materials analyzed.

An important observation is the aluminum concentration found in the sludge from the Gurjaú WTP, which was lower than expected. In our sample, converting the result format, the XRF analysis indicated 5.07% Al_2_O_3_, whereas values reported in the literature for WTPS generally fall within a higher range, typically between 14.38% and 40% Al_2_O_3_ (Richter 2001; Lucena et al. 2016; Ahmad et al. 2016; Ciuła et al. 2024; Reis et al. 2024). Additionally, two studies reporting aluminum directly in elemental form (as Al) for WTPS describe concentrations of 118,700, 141,000, and 147,000 mg·kg^−1^ (Peters and Basta 1996; Ippolito et al. 2011). These data show the variability in the composition of WTPS and such discrepancies are consistent with the well-known dependence of aluminum content on multiple operational and environmental factors, including the coagulant dosage applied, the type and operational configuration of the treatment plant, and the quality of the raw water. This deviation with the results in the literature may also be attributed to the exposure of the dewatered sludge to rainfall, which likely favored aluminum leaching.

This chemical loss directly affects the properties of the extracts generated from the sludge. In the case of the synthesized zeolite, a Si/Al ratio of 2.20 was observed, which falls within the expected range. Table 1, presented below, summarizes the chemical constituents of the samples, highlighting the major elements and their average concentrations along with the respective standard deviations.

**Table 1:** Chemical constituents (mg kg^−1^) of SCBA, WTPS, and zeolitic compound determined by XRF

Elements	SCBA (mg·kg^−1^)	SD	WTPS (mg·kg^−1^)	SD	Zeolitic compound (mg·kg^−1^)	SD
Si	183,010	222	161,750	212	275,000	347
Al	41,755	266	70,915	454	120,000	725
Fe	29,317	103	49,402	134	423	18
Ca	16,880	57	622	17	852	24
Mg	10,661	1530	4224	1109	1834	742
K	15,103	65	1841	31	236	32
Ti	3191	24	5556	28	< LD	
Mn	640	9	113	7	< LD	
P	741	21	280	18	319	27
Zr	338	3	34	2	5	3
Zn	277	7	61	4	54	4
Cu	80	5	29	4	48	5
Ni	16	5	10	5	< LD	
Cr	12	6	99	6	< LD	
Pb	12	5	45	6	223	11
LOI	11.16%		20.32%		8.47%	

*SD* standard deviation, *LOI* loss on ignition, *LD* limit of detection

#### X-ray diffraction

**Fig. 1:**
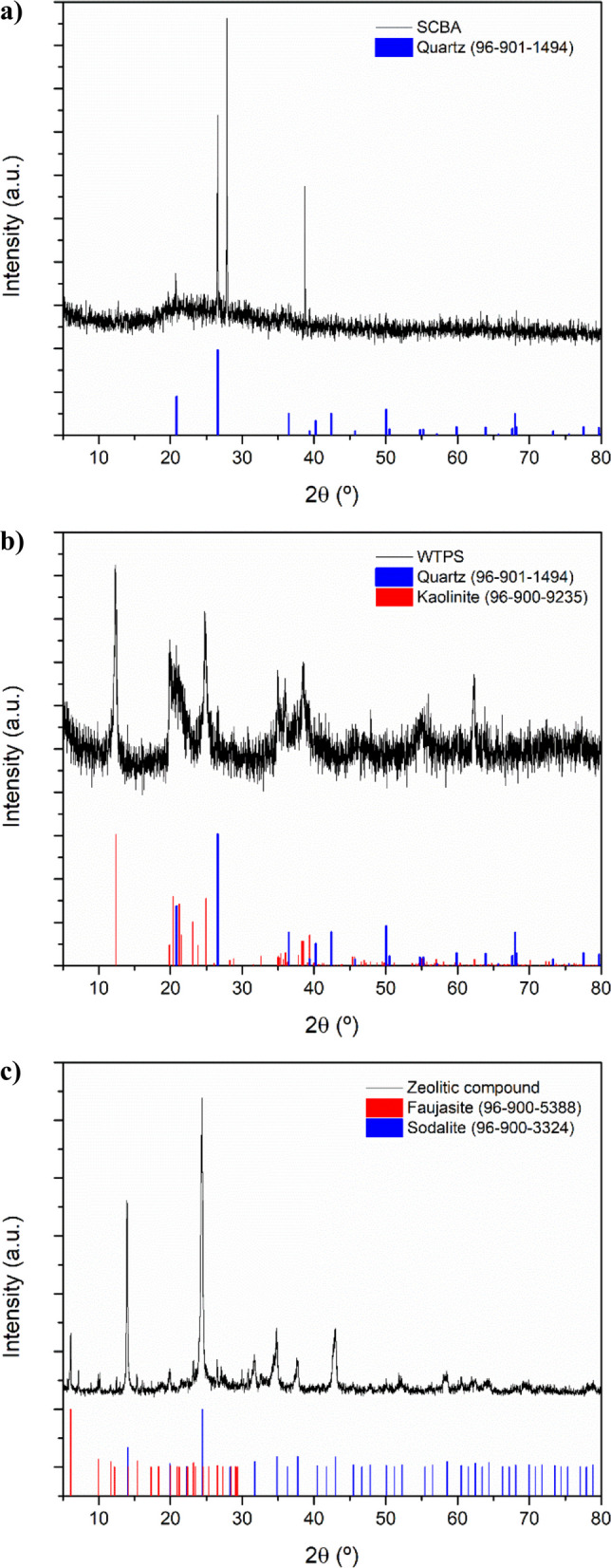
**a** XRD profile of SCBA along with the standard XRD data for quartz; **b** XRD profile of WTPS along with the standard XRD data for quartz and kaolinite; **c** XRD profile of zeolitic compound along with the standard XRD data for faujasite and sodalite

In the XRD pattern of SCBA (Fig. 1a) indicating the presence of quartz (α-SiO_2_) when compared to its reference pattern (COD 96–901-1494). These results corroborate the XRF analysis, which identified silicon as the predominant element, a fact also observed in previous studies (Ribeiro and Morelli 2014; Patel 2020), which also characterized SCBA. The high quartz content is attributed to the presence of sand adhered to the sugar cane or to the quartz itself present in the fibrous residues of the plant (Jagadesh et al. 2015).

The WTPS diffractogram shown in Fig. 1b indicates that the sample is partially crystalline, as the peaks are well-defined, although somewhat broadened. Comparison with known crystalline standards (represented by the colored lines at the bottom of the graph) confirms the presence of quartz (COD 96–901-1494) and kaolinite (COD 96–900-9235). Moreover, the elevated background and the diffuse character of some regions of the spectrum suggest the existence of an amorphous phase mixed with the crystalline phases, which can be attributed to the presence of organic matter in the sample (Mitchell 2003; Fan et al. 2015).

The X-ray diffraction pattern of the zeolitic compound is shown in Fig. 1c. The main crystalline materials identified are two types of zeolite: sodalite (COD 96–900–3324) and faujasite (COD 96–900–5388), with sodalite being the predominant phase, at a calculated ratio of 95:5 relative to faujasite.

#### Infrared spectroscopy analysis

**Fig. 2:**
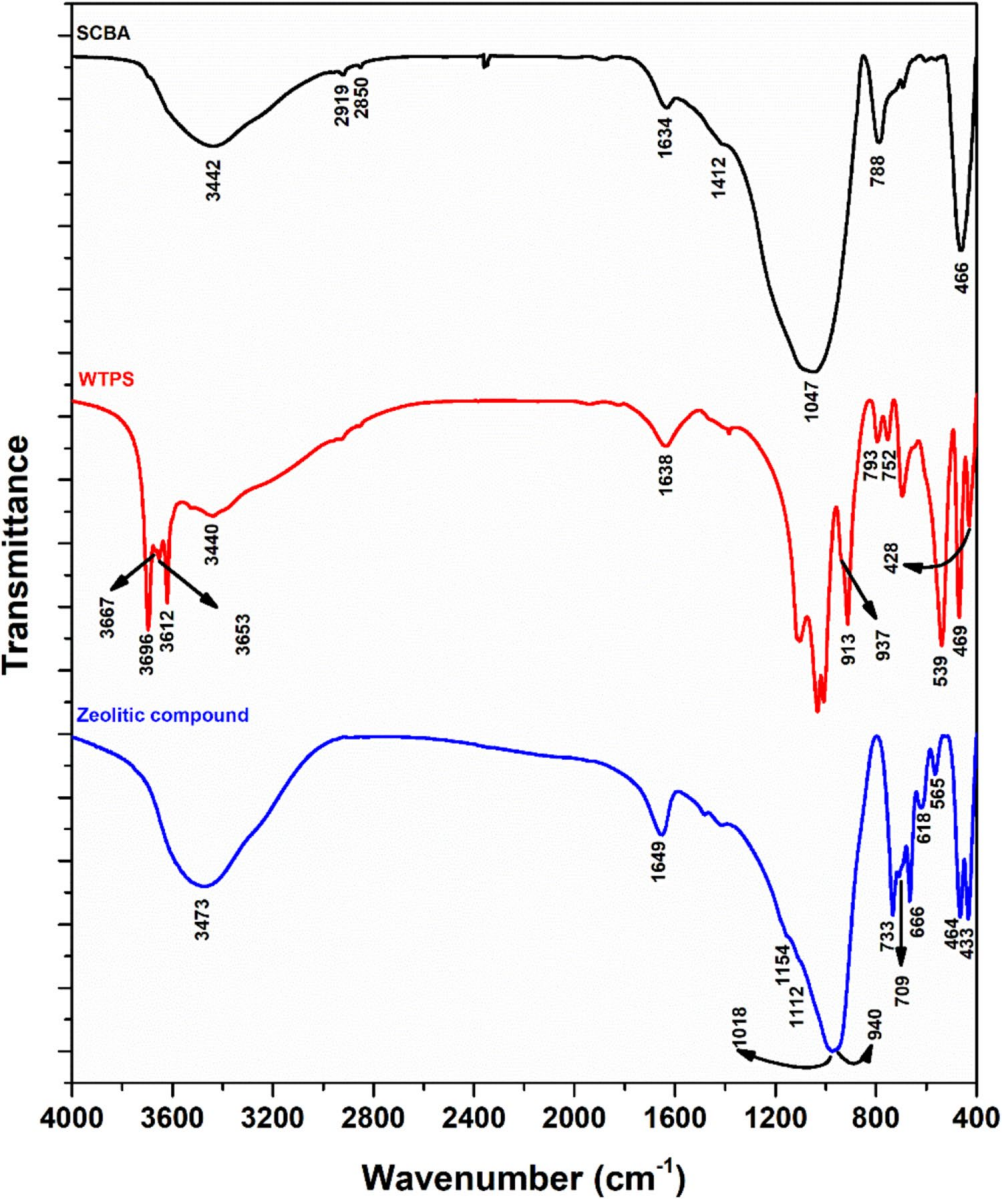
Infrared spectra of SCBA, WTPS, and zeolitic compound samples

Infrared spectroscopy studies contribute to the identification of the main functional groups present in the compounds. The analyses of the SCBA, WTPS, and zeolitic compound samples are shown in Fig. 2.

In the SCBA spectrum, a broad absorption band observed in the 3750–3000 cm^−1^ region may be attributed to the O–H stretching vibrations of hydroxyl groups associated with carbonaceous materials and silicates (Abdelkhalek et al. 2022; Pereira et al. 2024). The absorption bands observed at 2850 and 2919 cm^−1^ are attributed to the symmetric and asymmetric stretching vibrations of the -CH_2_ functional group, respectively (Zhu et al. 2016). The absorption at 1634 cm^−1^ is related to the symmetric bending (“scissoring”) of H–O-H bonds in molecular water (Lee and Lee 2015). The band at 1412 cm^−1^ corresponds to the C = C stretching of aromatic rings polarized by oxygen atoms bonded to one of the carbon atoms, as found in lignin (Salman et al. 2011). Silverstein et al. (2005) indicate that Si–O absorption bands typically occur in the 830–1110 cm^−1^ region, aligning with the 1047 cm^−1^ band identified in this study. The signal observed near 788 cm^−1^ is associated with the symmetric stretching of Si–O–Si bonds, a feature commonly found in quartz structures (Yan et al. 2012; Capeletti and Zimnoch 2016). The band around 466 cm^−1^ corresponds to SiO_4_ tetrahedral bending vibrations and is likewise indicative of quartz presence (Król et al. 2012; Jovanovski and Makreski 2016). These strong absorption bands support the evidence of a high silica content in the SCBA.

Regarding the WTPS analysis, the groups identified in the spectrum suggest a strong presence of characteristic kaolinite bands (Vahur et al. 2016). In the FTIR spectrum of the WTPS, bands were observed at 3696, 3667, 3653, and 3620 cm^−1^, associated with OH groups; the band at 3696 cm^−1^ is related to symmetric stretching vibration, while the absorptions at 3667 and 3653 cm^−1^ can be attributed to out-of-plane stretching vibrations; and the absorption near 3620 cm^−1^ is assigned to internal hydroxyl groups (Maia et al. 2014; Vahur et al. 2016). A band around 3440 cm^−1^ may indicate the presence of structural Fe in kaolinite (Maia et al. 2014). The deformation vibrations of adsorbed water were observed at 1638 cm^−1^, and several strong, well-resolved bands in the 1120–1000 cm^−1^ range correspond to Si–O stretching vibrations in kaolinite (Jozanikohan and Abarghooei 2022). Bands located at 937 and 913 cm^−1^ were observed and are consistent with Al-Al–OH deformation bands (Ling et al. 2017; Davarcioglu 2011). The absorption bands at 793 and 752 cm^−1^ are attributed to symmetric Si–O–Si stretching vibrations of quartz, while those at 469 and 428 cm^−1^ are characteristic of Si–O bending vibrations, specific to SiO_4_ tetrahedra in quartz (Paz et al. 2010; Jovanovski and Makreski 2016). A band at approximately 539 cm^−1^ was observed, associated with the Al(O,OH)₆ octahedron (Maia et al. 2014).

In the zeolitic compound spectrum, the presence of a band at 3473 cm^−1^, associated with O–H stretching vibrations, may be attributed both to the hydration of aluminosilicates and to the presence of surface -T-OH groups (T = Si, Al) on the zeolite (Król et al. 2016; Han et al. 2019). Additionally, the band at 1649 cm^−1^, related to H–O-H bending vibrations, confirms the presence of zeolitic water in the resulting samples (Melo et al. 2017; Yao et al. 2018). The asymmetric and symmetric T-O-T stretching vibrations of TO_4_ tetrahedra are characteristic of zeolitic materials and appear in the wavenumber ranges of 950–1250 cm^−1^ and 650–750 cm^−1^, respectively (Sriatun et al. 2018; Han et al. 2019). These are represented in the FTIR spectrum of the zeolite by the bands at approximately 1154, 1112, 1018, 709, 733, and 666 cm^−1^. The presence of a band at 940 cm^−1^ may be attributed to T-OH stretching vibrations (Faghihian et al. 2012; Yao et al. 2018). The bands at 565 and 618 cm^−1^ correspond to collective vibrations of structural rings of TO_4_ tetrahedra, typical of the 500–650 cm^−1^ region in zeolite FTIR spectra (Ma and Lothenbach 2021). Finally, the bands at 464 and 433 cm^−1^ can be attributed to O-T-O bending vibrations (Bellatreccia et al. 2009), indicating the presence of highly crystalline aluminosilicate products, also confirmed by the XRD spectrum.

#### Scanning electron microscopy

**Fig. 3:**
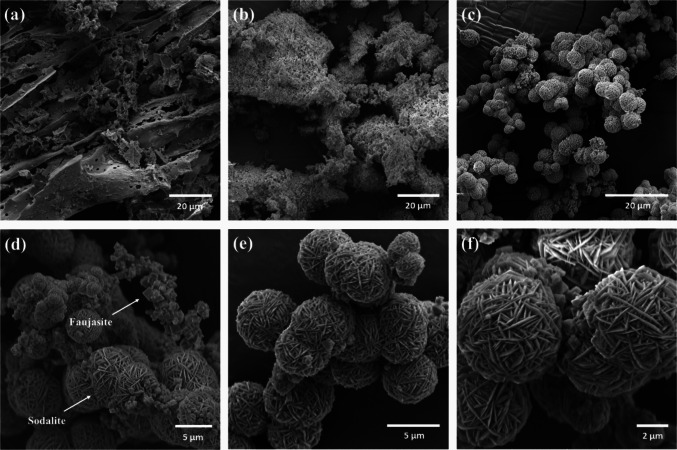
SEM images showing **a** SCBA, **b** WTPS, **c** synthesized zeolitic compound, and SEM images of the synthesized zeolitic compound at different magnifications: **d** 7000 × , **e** 10,000 × , and **f** 15,000 ×

As illustrated in Fig. 3a, SCBA’s morphology is inherently macroporous, directly linked to the cavitous, fibrous structure of the parent sugarcane bagasse. The fiber-like elements are clearly visible, highlighting this porosity. Conversely, SEM imaging of WTPS (Fig. 3b) reveals agglomerated particles with irregular and varied shapes, demonstrating a lack of uniform size distribution and a non-macroporous architecture. Figure 3c visually confirms the mixed morphology of the synthesized zeolite, displaying both octahedral particles, typical of Faujasite (Zeolite X) (Krachuamram et al. 2021), and the spherical “wool ball” aggregates associated with the dominant Sodalite phase (Sousa et al. 2020; E 2017). Figures 3d-f provide further detailed SEM views of the zeolitic compound at higher magnifications.

#### Thermogravimetric analysis

**Fig. 4:**
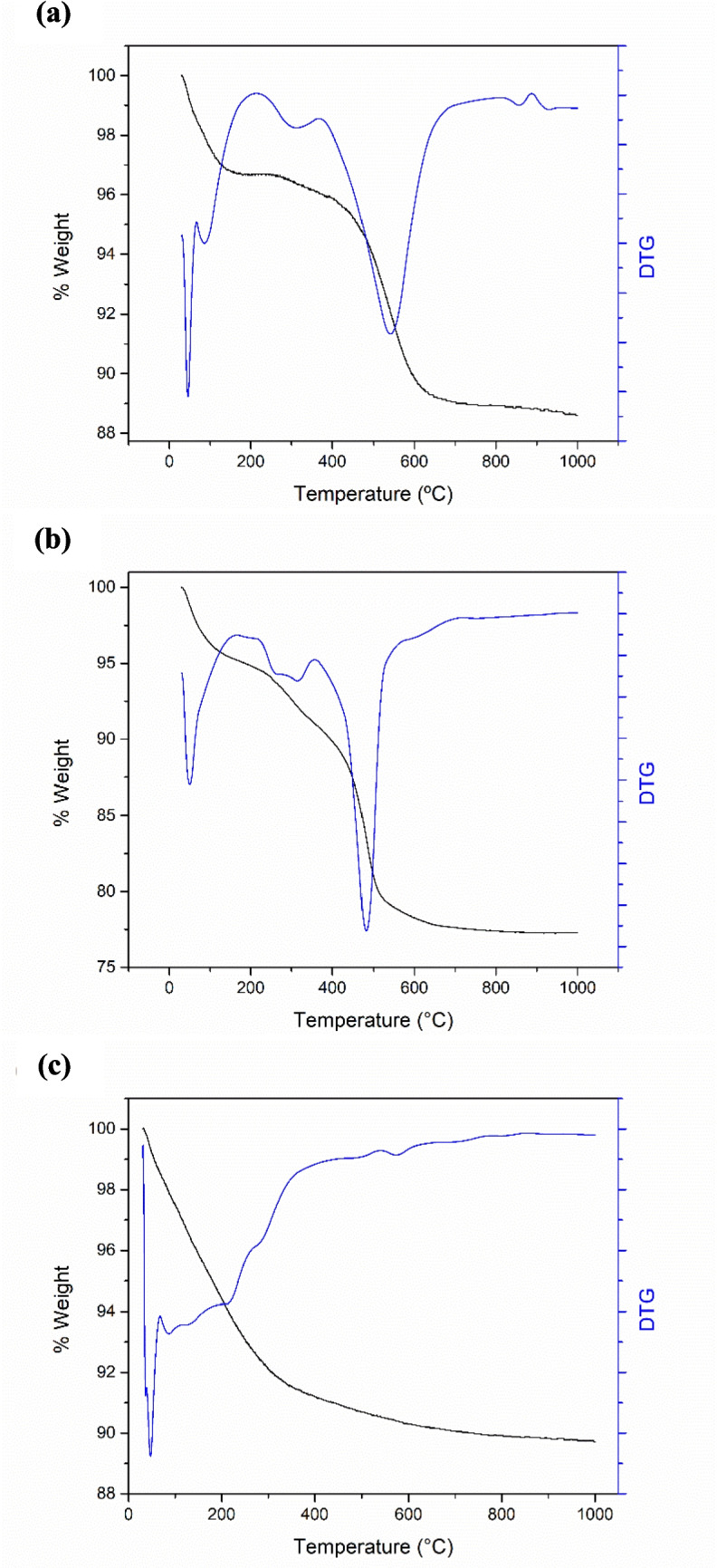
TGA/DTG curve of **a** SCBA; **b** WTPS; **c** Zeolitic compound

The TGA/DTG profiles for the SCBA and WTPS materials are presented in Figs. 4a and b, respectively. The first two thermal events in the curves are associated with the loss of water content and volatile compounds present in the samples. Subsequently, the last two events are attributed to pyrolysis and oxidation, resulting in a total degradation of 22.72% for the WTPS and 11.41% for the SCBA. These results are consistent with the data obtained from the XRF analysis, and it is important to highlight that both samples contain organic matter. In the SCBA, this organic matter originates from the incomplete combustion of bagasse in the boilers (Sakib et al. 2023). In the WTPS, the presence of organic matter arises from solids removed during the water treatment process. Presented in Fig. 4c are the TGA and DTG curves for the synthesized zeolite. The weight losses at temperatures below 500 °C are attributed to the removal of physically adsorbed water physically adsorbed on the surface and the loss of zeolitic water, which is retained within the material’s matrix (Ngoc et al. 2013).

#### Textural characterization by nitrogen adsorption isotherm

The textural analysis using nitrogen adsorption isotherms was performed to determine the specific surface area and pore characteristics of the materials, with the values presented in Table 2. The specific surface area of SCBA was 22.92 m^2^·g^−1^, a relatively low value; however, it is important to note that the ashes were used without any activation treatment. Regarding the WTPS, the value of 48.39 m^2^·g^−1^ is close to the range described in other studies, which varies from 61.58 to 83.2 m^2^·g^−1^ (Shalaby et al. 2017; Siswoyo et al. 2019; Tony 2020). As for the synthesized zeolitic compound, the surface area was 11.72 m^2^·g^−1^. Although this value is low, it falls within the range reported in the literature for the predominant sodalite phase, which varies from 2.00 to 43.34 m^2^·g^−1^ (Shanbhag et al. 2009; Ji et al. 2011; Hiyoshi 2012; Franus et al. 2014; Kaminishikawahara et al. 2015; Gilani et al. 2021; El-Kordy et al. 2022). Regarding the average pore diameter, the values obtained from the adsorption analysis (BJH method) indicate the presence of mesopores, located near the lower limit of the classification proposed by IUPAC (2–50 nm) (Rouquerol et al. 1994). The total pore volume, estimated at 6.25 × 10^−3^ cm^3^·g^−1^ for the zeolite and calculated near saturation (*P*/*P*_o_ ≈ 0.99), suggests a reduced overall porosity, which may be associated with the presence of very narrow pores with limited access.

**Table 2:** Data on the properties of the porous structure of the precursor materials and the synthesized adsorbents

	***S***_**BET**_** (m**^**2**^**·g**^**−1**^**)**	**Average pore diameter (nm)**	**Total pore volume (cm**^**3**^**·g**^**−1**^**)**
SCBA	22.92	4.353	2.49 × 10^−2^
WTPS	48.39	5.496	6.65 × 10^−2^
Zeolitic compound	11.72	2.133	6.25 × 10^−3^

*S*_*BET*_ specific surface area BET

#### Point of zero charge

**Fig. 5:**
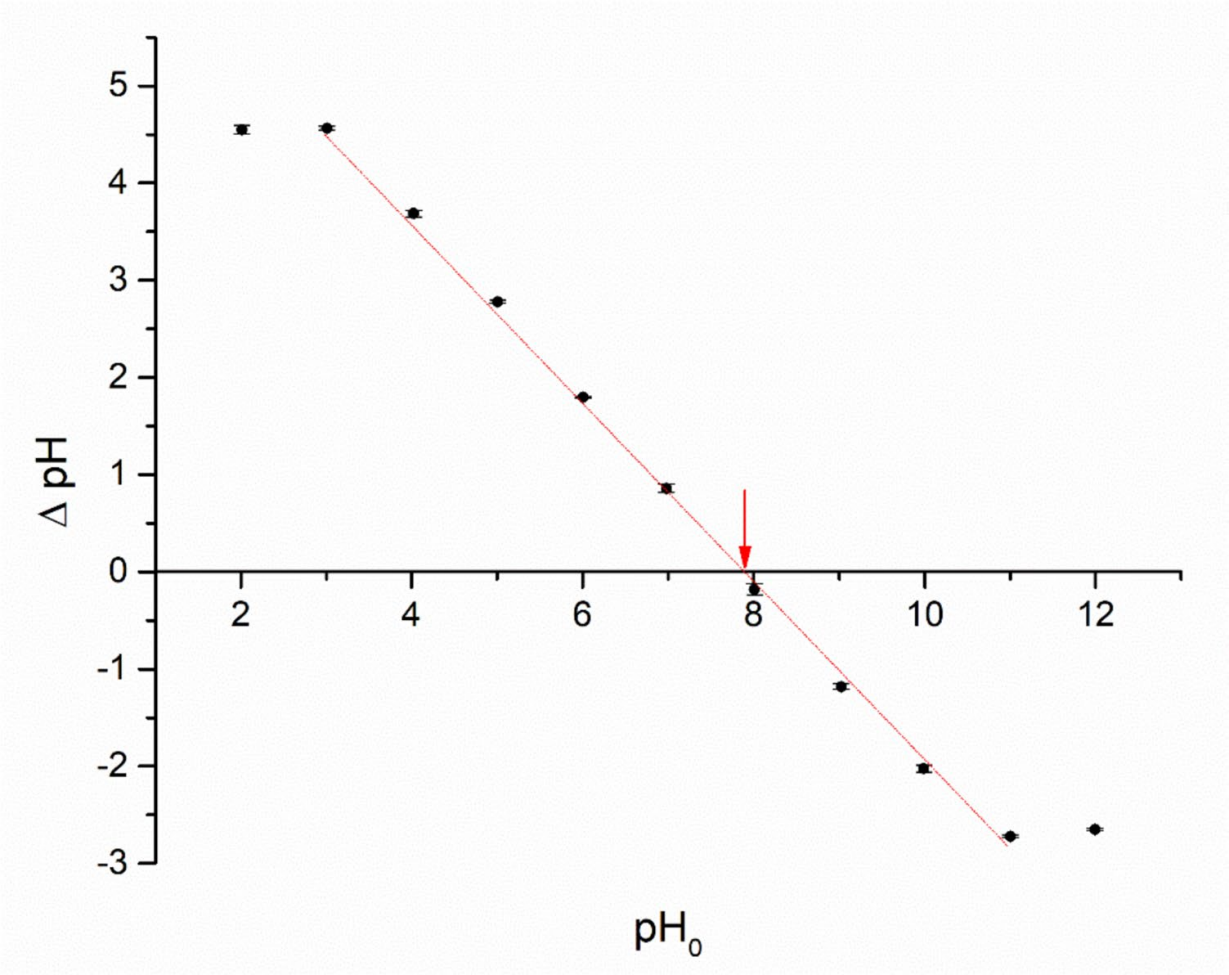
Determination of pHₚ_zc_ for zeolitic compound

The point of zero charge (pHₚ_zc_) refers to the pH at which the surface exhibits equal amounts of positive and negative charges, resulting in a net zero surface charge. This parameter is specific to a given solid surface in a particular aqueous medium (electrolyte) (Rey et al. 2017). Knowing the pHₚ_zc_ is essential to anticipate the ionization behavior of functional groups present on the surface and how they interact with dissolved species. When the pH is below the pHₚ_zc_, the material’s surface tends to be positively charged, which enhances the adsorption of anionic species (Freitas et al. 2015). A plot of ΔpH against initial pH for the synthesized zeolitic compound was obtained (Fig. 5), and the point of intersection with the *x*-axis—i.e., where ΔpH = 0—corresponds to the pHₚ_zc_. The pHₚ_zc_ value for the zeolitic compound was 7.91, indicating the pH at which the zeolite’s surface charge is neutral.

### Extract characterization

Table 3 presents the concentrations of aluminum, silicon, sodium, manganese, and copper in the extracts obtained from SCBA and WTPS. Since the same extraction process was applied to both samples, the observed differences between the extracts mainly reflect variations in the chemical composition of the original matrices.

**Table 3:** Concentration in mg·L^−1^ of silicon, sodium and some metals in SCBA and WTPS extracts

	Al (mg·L^−1^)	Si (mg·L^−1^)	Na (mg·L^−1^)	Mn (mg·L^−1^)	Cu (mg·L^−1^)
Extract from SCBA	570.22 ± 174.34	17,077.06	9.29 × 10^4^ ± 1.52 × 10^4^	17.80 ± 0.72	95.68 ± 25.9
Extract from WTPS	3007.50 ± 120.30	1861.86	9.33 × 10^4^ ± 1.68 × 10^4^	< 8.02	104.5 ± 61.31

It can be seen that the aluminum concentration in the WTPS extract (3007.50 ± 120.30 mg·L^−1^) is significantly higher than in the SCBA extract (570.22 ± 174.34 mg·L^−1^). This behavior is directly related to the greater presence of aluminum in the WTPS matrix (70,915 mg·kg^−1^) compared to the SCBA (41,755 mg·kg^−1^). In contrast, silicon shows the opposite behavior, with a much higher concentration in SCBA (17,077.06 mg·L^−1^) than in WTPS (1861.86 mg·L^−1^). Although the silicon concentrations in the SCBA (183,010 mg·kg^−1^) and WTPS (161,750 mg·kg^−1^) matrices are similar, the difference in the extracts suggests that the silicon in SCBA is in a more soluble form or more readily available for extraction than in WTPS, possibly due to differences in mineralogy and the chemical bondings of this element in each matrix (Schaller et al. 2021; Li et al. 2022).

The sodium concentration is similar in both extracts, which can be attributed to the fact that this element was added during the extraction process in equivalent amounts for both matrices. Regarding manganese, a significant difference can be observed between SCBA (17.80 ± 0.72 mg·L^−1^) and WTPS (< 8.02 mg·L^−1^), which can be explained by the initial metal content in the matrices (640 mg·kg^−1^ in SCBA versus 113 mg·kg^−1^ in WTPS). As for copper, although SCBA has a significantly higher copper concentration (80 mg·kg^−1^) compared to WTPS (29 mg·kg^−1^), the values obtained in the extracts are similar. This suggests that factors such as differences in copper solubility or its interaction with other components of the extraction medium may have influenced the recovery of this element.

Another important point is the Si/Al ratio in the extracts, which can influence their applicability. In SCBA, this ratio is high due to the elevated silicon concentration, while in WTPS, the higher aluminum concentration reduces this value. Initially, it was intended to mix the two extracts to achieve a Si/Al ratio of around 2. However, given the concentrations observed in the extracts, this approach was not sufficient to reach the desired ratio.

Therefore, it was decided to add aluminum sulfate to supplement the necessary amount of aluminum. This choice is justified by the fact that aluminum sulfate is a cost-effective alternative compared to other aluminum sources commonly used in zeolite synthesis, such as sodium aluminate and aluminum isopropoxide (Moisés et al. 2013; Bing et al. 2020; Araújo Filho et al. 2021), and it has already been employed as the sole aluminum source in this type of process (Kamali et al. 2009).

While the core objective of this work is the maximum valorization of residues and the consequent reduction in the use of commercial reagents, the inherent compositional variability of waste materials makes it highly probable that supplementation with small amounts of commercial inputs will be necessary to achieve the precise stoichiometric ratios required for successful and reproducible zeolite synthesis.

In this context, although the dual-residue approach proved conceptually viable, relying solely on the aluminum extracted from WTPS would require using a proportionally large volume of this extract to reach the targeted Si/Al ratio of ~ 2, initially selected to guide the synthesis conditions. Such an adjustment would excessively dilute the reaction mixture, potentially lowering the synthesis efficiency. To avoid this and to ensure tighter control over the stoichiometry, a small amount of aluminum sulfate was added to facilitate achieving the desired Si/Al ratio and more concentrated and favorable reaction conditions.

The addition of a minimal amount of aluminum sulfate thus ensured that the process could proceed effectively without compromising the sustainability of the route. Importantly, the proposed method remains centered on the valorization of two industrial residues, distinguishing this study from previous works that rely exclusively on a single waste combined with commercial reagents. This adjustment reflects a practical and scalable solution for implementing waste-based zeolite synthesis under real-world conditions.

### Adsorption results

#### Influence of pH on dye adsorption

**Fig. 6:**
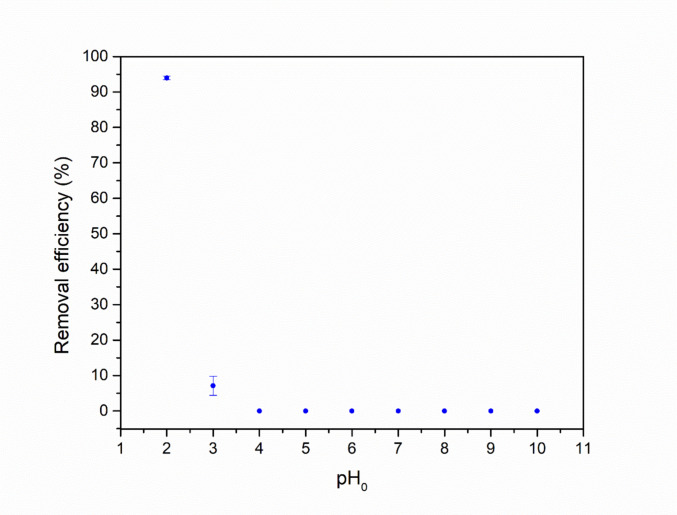
Influence of pH on dye adsorption efficiency (conditions applied: 30 mg·L^−1^ of initial dye concentration, 4 g·L^−1^ of adsorbent, agitation at 300 rpm, temperature fixed at 25 ± 1 °C, and contact time maintained until equilibrium was achieved)

Figure 6 shows the effect of solution pH on the adsorption of AR27 onto the zeolitic compound at various initial pH values. As shown in this figure, the removal efficiency increased with the extreme decrease of the initial solution pH, reaching its maximum at pH 2, and becoming practically null above pH 4. At pH 2, the positive charge of the zeolite results from the protonation of surface functional groups, such as aluminol groups (–Al–OH) and, to a lesser extent, silanol groups (–Si–OH). The AR27 dye is an anionic azo dye containing three sulfonic groups (R–SO_3_Na), which, in aqueous solution, dissociate into sodium ions (Na⁺) and sulfonate anions (–SO_3_^−^) (Fiorentin et al. 2010; Scheufele et al. 2016). Although in acidic media these groups could theoretically be protonated (R–SO_3_H), sulfonic groups maintain a negative charge even in strongly acidic environments, due to their estimated pKa values being lower than zero (Errais et al. 2012; Konicki et al. 2015). Thus, the proton-rich environment promotes the protonation of the zeolite surface, increasing its positive charge and favoring the electrostatic attraction of the dye anions.

It is also noteworthy that UV–Vis spectral analysis revealed no shift in the characteristic absorption peak of AR27 with pH variation in the studied range (2–10), indicating that the electronic structure of the dye remained stable across all tested conditions (Fig. S9, Section S3 of the supplementary material). Therefore, the observed changes in removal efficiency are not attributed to chemical modifications of the dye, but rather to interactions between the dye and the surface of the adsorbent.

These results are consistent with the point of zero charge (pHₚ_zc_ = 7.91) of the zeolitic compound. Since the pH range in which significant adsorption was observed is below the pHₚzc, the positive surface charge of the material favored the interaction with the anionic dye, reinforcing the importance of surface charge in adsorption processes.

#### Effect of varying adsorbent dosage

**Fig. 7:**
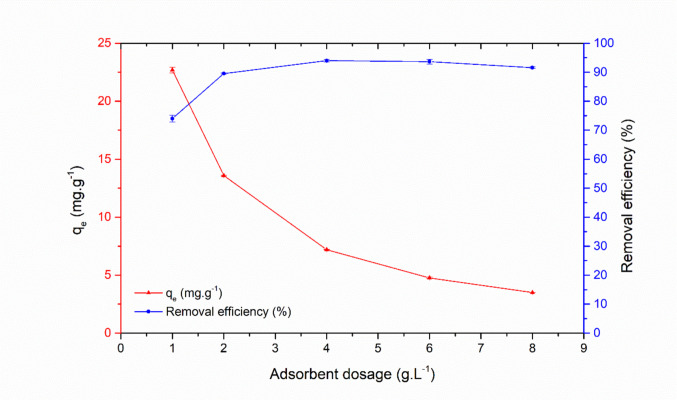
Influence of the amount of zeolitic compound on AR27 dye adsorption (Conditions applied: 30 mg·L^−1^ of initial dye concentration at pH_0_ 2, agitation at 300 rpm, temperature fixed at 25 ± 1 °C, and contact time maintained until equilibrium was achieved)

Based on Fig. 7, it can be observed that dye removal increased as the zeolite dosage was raised, reaching its maximum at 4 g·L^−1^, followed by a slight decrease. This initial behavior can be attributed to the greater availability of surface area and active sites with the increasing amount of adsorbent. However, beyond this point (4 g·L^−1^), a slight decrease in removal efficiency was initially attributed to particle agglomeration, which reduces the effective surface area accessible for adsorption (Hor et al. 2016; Shakoor and Nasar 2016). Nevertheless, a statistical analysis (one-way ANOVA) revealed that the differences among the highest dosages were not statistically significant (*p* > 0.05), suggesting that the observed decrease may fall within the margin of experimental variation. Additionally, when evaluating the adsorptive capacity, a decrease was observed with increasing adsorbent dosage, resulting in lower efficiency per unit mass. Therefore, considering both the statistical similarity in removal at higher dosages and the need to optimize adsorbent use, a dosage of 1.5 g·L^−1^ was selected as the ideal condition for subsequent experiments.

#### Adsorption equilibrium

**Fig. 8:**
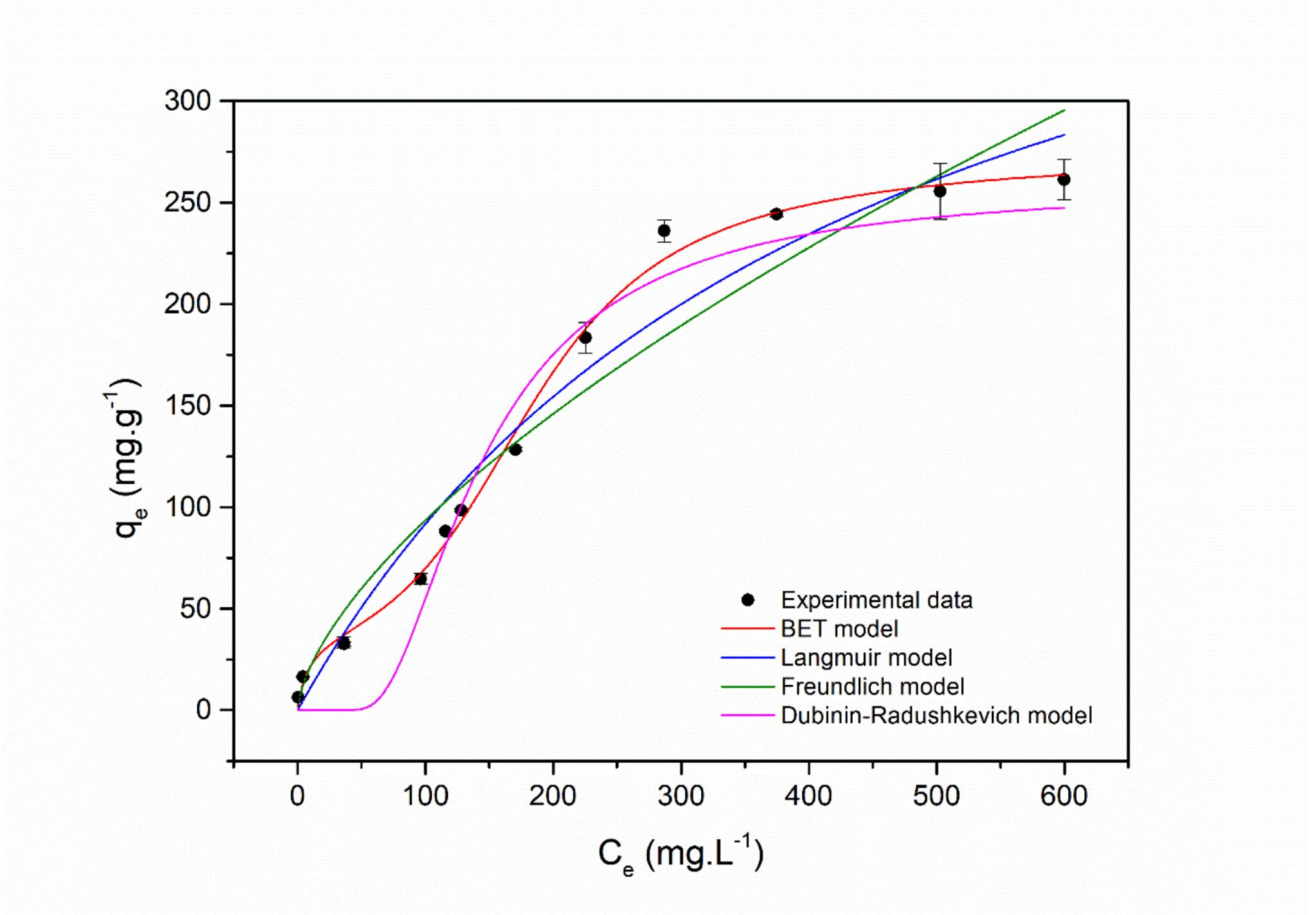
Adsorption equilibrium isotherms of AR27 onto the zeolitic compound. (Conditions applied: initial dye concentrations ranging from 10 to 1000 mg·L^−1^ at pH_0_ 2, 1.5 g·L^−1^ of adsorbent, agitation at 300 rpm, temperature fixed at 25 ± 1 °C, and contact time maintained until equilibrium was achieved)

The adsorption isotherm of Acid Red 27 onto the zeolite was analyzed using the Langmuir, Freundlich, and BET models. The fitted parameters of these models are presented in Table 4, while Fig. 8 illustrates the models’ fits to the experimental data.

**Table 4:** Isotherm model parameters for AR27 adsorption onto the zeolitic compound, including the RSS, *R*^2^, and *χ*^2^ values derived from nonlinear fitting (Conditions applied: initial dye concentrations ranging from 5 to 1000 mg·L^−1^ at pH_0_ 2, 1.5 g·L^−1^ of adsorbent, agitation at 300 rpm, temperature fixed at 25 ± 1 °C, and contact time maintained until equilibrium was achieved)

Adsorption isotherm	Parameter	Value
***Langmuir***	*q*_*max*_ (mg·g^−1^)	497.1239 ± 66.0441
	*K*_*L*_ (L·mg^−1^)	2.22 × 10^−3^ ± 5.1241 × 10^−4^
	RSS	4483.0289
	*R*^2^	0.9689
	*χ*^2^	298.8686
***Freundlich***	*K*_*F*_ ([(mg·g^−1^).(mg·L^−1^)^−1/n^])	4.3125 ± 1.5089
	*n*	1.5114 ± 0.1384
	RSS	7287.0837
	*R*^2^	0.9495
	*χ*^2^	485.8056
***BET***	*q*_*m*_ (mg·g^−1^)	35.0106 ± 0.4937
	*K*_*S*_ (L·mg^−1^)	0.1875 ± 0.0710
	*K*_*L*_ (L·mg^−1^)	5.26 × 10^–3^ ± 1.30 × 10^−4^
	*n*	8
	RSS	419.6429
	*R*^2^	0.9971
	*χ*^2^	29.9745
***Dubinin–Radushkevich***	*K*_DR_ (mol^2^·kJ^−2^)	2.54 × 10^–3^ ± 9.14 × 10^–4^
	*q*_DR_ (mg·g^−1^)	258.4066 ± 64.9622
	*E*_DR_ (kJ·mol^−1^)	0.014 kJ·mol^−1^
	RSS	20,976.6641
	*R*^2^	0.7324
	*χ*^2^	1906.9695

The analysis of adsorption isotherm models for the AR27 dye onto the zeolite revealed that the BET model provided the best fit to the experimental data. The BET model achieved an *R*^2^ of 0.9971, significantly higher than the values obtained for the Langmuir (0.9689) and Freundlich (0.9495) models. Moreover, the chi-square factor (*χ*^2^) value for the BET model was 29.97, considerably lower than the values of 298.87 for Langmuir and 485.81 for Freundlich. The lower *χ*^2^ value indicates that the differences between the experimental data and the model-predicted values are less significant, suggesting a more accurate fit. Similarly, the RSS value for the BET model (419.64) was also lower than those of the other models, further supporting the model’s suitability for describing the experimental data.

The better fit of the BET model may be related to the multilayer formation during the adsorption of AR27 dye onto the zeolite, combined with the heterogeneity of the adsorbent surface. The Langmuir model, on the other hand, assumes monolayer adsorption on a homogeneous surface with a finite number of equivalent sites (Langmuir 1918; Bensalah 2024). The Freundlich model, although considering a heterogeneous surface and allowing for multilayer adsorption in an empirical manner, does not explicitly describe the sequential growth of layers as proposed by the BET theory (Hu et al. 2024; Liu et al. 2010; Aziam et al. 2024). The BET theory allows for the continuous adsorption of molecules onto previously adsorbed layers, driven by intermolecular interactions, thus extending the process beyond the occupation of the first active sites (Yahia et al. 2013; Rahman et al. 2019). The isotherm graphs (Fig. 8) describe this assumption, showing the BET model (red line) following the experimental data points more closely across the entire concentration range of the dye, whereas the Langmuir and Freundlich models exhibit deviations in certain regions.

In the BET isotherm equation, the monolayer adsorption capacity (*q*_*m*_) differs from the maximum adsorption capacity, which is limited due to the assumption that the number of adsorbed layers cannot exceed a finite value *n* (Ebadi et al. 2009). The monolayer adsorption capacity is approximately 35 mg·g^−1^, indicating good retention on the first layer. However, adsorption continues beyond this phase, reaching a capacity of about 250 mg·g^−1^. The equilibrium constants indicate that adsorption on subsequent layers is less favorable than on the first layer, probably due to decreasing intermolecular interactions as new layers form. The number of layers (*n* = 8) reinforces the applicability of the BET model, showing that the process extends beyond a monolayer, albeit with lower efficiency in the upper layers. The low affinity for the subsequent layers is evidenced by the reduced value of the equilibrium constant (*K*_*L*_ = 5.26 × 10^−3^ L·mg^−1^), which is significantly lower than the constant for the first layer (*K*_*S*_ = 0.1875 L·mg^−1^). This difference indicates the lower intensity of intermolecular interactions in the upper layers and reinforces the stronger interaction between the dye and the active sites of the zeolite in the formation of the monolayer.

This behavior reinforces the uniqueness of the results obtained in this study, especially because no reports were found in the literature where the BET isotherm model was successfully applied to adsorption systems using zeolites as adsorbents and dyes as adsorbates. In the studies reviewed, including those by Fungaro et al. (2009), Saputra et al. (2017), Imessaoudene et al. (2023), and Tubon-Usca et al. (2025), the Freundlich model was the most frequently adopted, suggesting adsorption on heterogeneous surfaces with multilayer formation, but without the detailed characterization of the number of layers as predicted by the BET equation. Thus, the data obtained here not only indicate the formation of multiple layers but also highlight the predominant role of the initial interaction between the dye and the active sites of the zeolite, revealing an adsorption dynamic distinct from what is reported in the literature.

The extremely low mean adsorption energy obtained from the Dubinin–Radushkevich model (*E* ≈ 0.014 kJ·mol^−1^) clearly indicates that the adsorption process is governed by weak physical interactions (Hu and Zhang 2019). Such a low *E* value reflects the dominance of non-specific electrostatic attractions and hydrogen bonding in an aqueous environment, rather than chemical bond formation. It should be noted that the D–R model provides an averaged energetic description and may underestimate local interaction energies on heterogeneous surfaces, as it is based on the Polanyi potential theory and assumes a specific (Gaussian) energy distribution rather than a homogeneous surface of adsorption sites (Edet and Ifelebuegu 2020; AlJaberi 2024). Consequently, this assumption may not fully capture the complex energetic heterogeneity commonly observed in many real adsorbent materials. Therefore, the D–R energy should be interpreted as an indicator of the absence of chemisorption, rather than as a direct measure of individual interaction strengths. Furthermore, as a result, the adsorption process is expected to be largely reversible, as commonly observed for physisorption-driven systems dominated by electrostatic interactions.

#### Kinetic study and influence of contact duration on adsorption

**Fig. 9:**
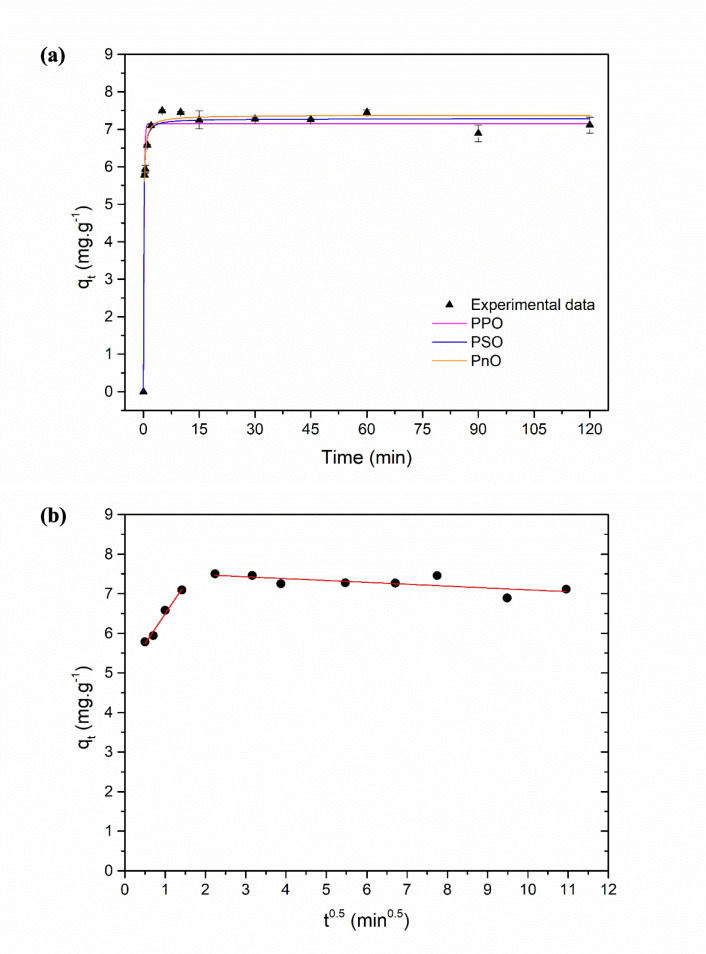
**a** Graphs of kinetic model fits (PFO, PSO, and PnO) for AR27 adsorption onto the zeolitic composite; **b** Weber–Morris intraparticle diffusion plot for AR27 adsorption (conditions applied: 30 mg·L^−1^ of initial dye concentration at pH_0_ 2, 4 g·L^−1^ of adsorbent, agitation at 300 rpm, temperature fixed at 25 ± 1 °C)

The kinetic behavior of Acid Red 27 removal by zeolite was investigated using the pseudo-first-order (PFO), pseudo-second-order (PSO), pseudo-nth-order (PnO), and Weber–Morris intraparticle diffusion model. The fitted parameters of all kinetic models are presented in Table 5, while Fig. 9a illustrates the fitting of the PFO, PSO, and PnO models to the experimental data, and Fig. 9b presents the Weber–Morris intraparticle diffusion plot.

**Table 5:** Kinetic parameters and statistical fit indices (RSS, *R*^2^, and *χ*^2^) for AR27 adsorption onto the zeolitic compound (conditions applied: 30 mg·L^−1^ of initial dye concentration at pH_0_ 2, 4 g·L^−1^ of adsorbent, agitation at 300 rpm, and temperature fixed at 25 ± 1 °C)

Kinetic model	Parameter	Value
Pseudo-first-order	Predicted *q*_*e*_(mg·g^−1^)	7.1514 ± 0.1149
	*k*_1_ (min^−1^)	5.5478 ± 0.7801
	RSS	1.4704
	*R*^2^	0.9697
	*χ*^2^	0.1337
Pseudo-second-order	Predicted *q*_*e*_(mg·g^−1^)	7.2832 ± 0.0798
	*k*_2_ (g·mg^−1^·min^−1^)	1.7486 ± 0.2872
	RSS	0.5899
	*R*^2^	0.9878
	*χ*^2^	0.0536
Pseudo-n-order	*q*_*max*_(mg·g^−1^)	7.3033 ± 0.1231
	*k*_*n*_(g^1−*n*^·mg^*n*−1^⋅min^−1^)	1.5296 ± 0.8730
	*n*	2.1338 ± 0.5309
	RSS	0.5826
	*R*^2^	0.8418
	*χ*^2^	0.0647
Intraparticle diffusion	*k*_*id*,1_ (mg·g^−1^·min^−0.5^)	1.5108 ± 0.1531
(Weber-Morris model)	*C*_1_ (mg·g^−1^)	4.9811 ± 0.1483
	RSS_1_	0.0221
	Pearson’s *r*_1_	0.9899
	*k*_*id*,2_ (mg·g^−1^·min^−0.5^)	−0.0471 ± 0.0188
	*C*_2_ (mg·g^−1^)	7.5690 ± 0.1287
	RSS_2_	0.1420
	Pearson’s *r*_2_	−0.7150

Based on the determination coefficients (*R*^2^), the pseudo-second-order (PSO) model showed the best fit to the experimental data (*R*^2^ = 0.9878), followed by the pseudo-first-order (PFO) model, with *R*^2^ = 0.9697. The pseudo-nth-order (PnO) model exhibited a lower performance (*R*^2^ = 0.8418). Although the PFO model achieved a reasonable fit, its performance was inferior to that of the PSO model. The PnO model also performed worse than the PFO model, suggesting that the additional flexibility of the PnO approach did not lead to a significant improvement in the fit. The PSO model also exhibited the lowest values of RSS (0.5899) and chi-square factor (*χ*^2^ = 0.0536), indicating that the residual error was smaller compared to the other models; the low *χ*^2^ value suggests that the differences between the model-predicted and observed values were minimal, reinforcing the adequacy of the fit. Thus, the PSO model describes the experimental data with higher precision compared to the other approaches analyzed.

This result suggests that the adsorption rate is governed by the availability and occupation of active sites located on the external surface of the zeolite (Hubbe et al. 2019). In that regard, the formation of the first adsorption layer of AR27 dye on the zeolitic composite is likely crucial and may limit the initial rate of the process, being slower despite being energetically favorable. This behavior may occur due to restrictions imposed by boundary layer diffusion, the need for molecular rearrangement to properly interact with active sites, and competition generated by the limited number of specific adsorption sites (Nasser et al. 2024; Shen et al. 2020). On the other hand, subsequent layers are formed more rapidly, although less stably, because they involve only intermolecular interactions. These interactions, although weaker, occur without significant diffusional barriers, as the molecules find a foundation in the first layer for adsorption, potentially facilitating the rapid formation of subsequent layers (Nasser et al. 2024). Therefore, the overall adsorption kinetics appear to be controlled by the energetic barrier associated with the formation of the first layer, while the subsequent adsorption stages proceed more swiftly.

This kinetic behavior is consistent with studies found in the literature that also used zeolites as adsorbents. Works such as those by Aysan et al. (2016), Gilani et al. (2021), and Tubon-Usca et al. (2025) have shown that the pseudo-second-order kinetic model was the most suitable for describing the adsorption of various dyes. These results indicate that, regardless of the origin or modification of the zeolite, the process tends to be governed by the number of available active sites and the chemical affinity between the adsorbent and the adsorbate.

Although the pseudo-second-order model provided the best statistical fit to the experimental kinetic data, it does not allow a direct identification of the mass transfer mechanisms involved in the adsorption process. Therefore, to further elucidate the contribution of diffusion phenomena and to verify whether intraparticle diffusion plays a controlling role in the adsorption of AR27 onto the zeolitic composite, the Weber–Morris intraparticle diffusion model was applied.

The Weber–Morris plot exhibited a multilinear profile, indicating that, according to Gül et al. (2019), the adsorption process occurs through more than one sequential stage rather than being governed by a single rate-controlling mechanism. The first linear region was described by the equation *q*_*t*_ = 1.5109 *t*^0.5^ + 4.9811, corresponding to the initial stage of adsorption. The relatively high value of the intraparticle diffusion constant (*k*_*id*,1_ = 1.5108 mg·g^−1^·min^−0.5^) suggests a rapid uptake of AR27 at the early stages, which can be attributed primarily to external mass transfer and surface adsorption (AlJaberi 2024). The positive intercept (C = 4.9812) indicates the presence of a significant boundary layer effect.

The second linear region was represented by the equation *q*_*t*_ =  − 0.0471 *t*^0.5^ + 7.5690, exhibiting an almost zero (slightly negative) slope. This behavior indicates that the system becomes dominated by equilibrium conditions, with negligible variation in adsorption capacity over time (Ji et al. 2024). The near-zero slope reflects the progressive saturation of active sites, while the slight negative value can be attributed to experimental fluctuations around the adsorption plateau.

Overall, the absence of a single linear region passing through the origin throughout the entire adsorption period demonstrates that intraparticle diffusion is not the dominant rate-controlling mechanism (Hubbe et al. 2019; Pap et al. 2025). Instead, both external mass transfer resistance and surface-related interactions significantly influence the adsorption kinetics.

For the higher concentrations (90, 500, and 1000 mg·L^−1^), the PSO model also provided the best fit. Similarly to what was observed for 30 mg·L^−1^, the Weber–Morris analysis for these higher concentrations indicated that intraparticle diffusion is not the rate-controlling step of the process. Detailed kinetic data for these concentrations are presented in Section S4 of the Supplementary Material.

The kinetic behavior of AR27 adsorption onto the zeolitic composite is best described by the pseudo-second-order model, indicating that the overall adsorption rate is governed by the availability and occupation of active sites and by the energetic interactions between the adsorbent and the adsorbate. The Weber–Morris analysis corroborates this interpretation by revealing a multilinear adsorption process and a significant boundary layer effect, demonstrating that surface-related processes dominate the adsorption kinetics, particularly during the initial stages. The combined kinetic analyses confirm that the adsorption of AR27 is primarily controlled by surface phenomena rather than by intraparticle diffusion, with the system rapidly approaching equilibrium as the active sites become progressively saturated.

#### Influence of anion coexistence

**Fig. 10:**
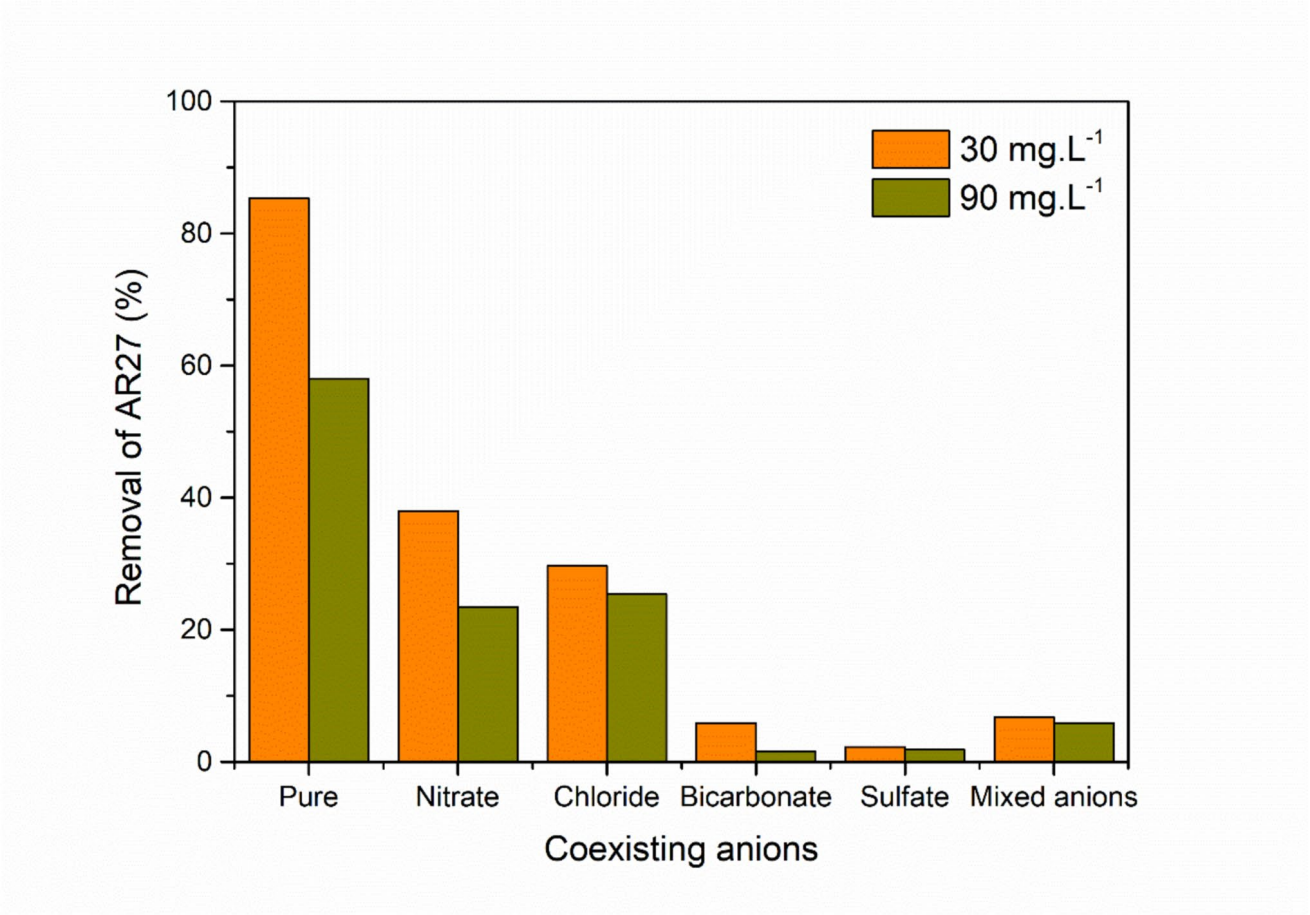
Influence of anion coexistence on AR27 removal by the zeolite compound (conditions applied: initial dye concentrations of 30 and 90 mg·L^−1^ at pH_0_ 2, coexisting anion concentration of 0.1 M, 1.5 g·L^−1^ of adsorbent, agitation at 300 rpm, temperature fixed at 25 ± 1 °C, and contact time maintained until equilibrium was achieved)

The adsorption of AR27 dye onto zeolite was evaluated in the presence of different coexisting anions (nitrate, chloride, bicarbonate, and sulfate), using solutions with initial dye concentrations of 30 mg/L and 90 mg/L (Fig. 10). It was observed that, in the absence of additional anions (pure condition), dye removal was the highest, reaching approximately 85% for the lower concentration solution and about 60% for the more concentrated solution. However, when anions were introduced into the system, a reduction in removal efficiency occurred, indicating competition between the ions in solution for the active sites on the zeolite.

Among the tested anions, sulfate and bicarbonate were the most significant inhibitors of AR27 adsorption. This phenomenon can be explained by the charge and interaction capacity of these anions with the zeolite surface. The sulfate ion, being divalent, exhibits a strong affinity for positively charged sites, directly competing with the anionic dye molecules. Its adsorption on the adsorbent surface is more favored compared to monovalent anions, due to the stronger electrostatic attraction (Li and Liu 2025). Bicarbonate, although monovalent, can influence the surface charge balance of the zeolite and specifically reduce adsorption by shifting the local equilibrium pH toward values less favorable for AR27 adsorption and by competing directly for the available active sites by electrostatic interactions (Tang et al. 2009; Derkani et al. 2019; Mohammed et al. 2021).

On the other hand, chloride and nitrate exerted a less pronounced inhibitory effect. This can be attributed to the fact that they are monovalent anions with a lower tendency for specific interaction with the active sites of the zeolite (Dong et al. 2018). As a result, competition with the dye is weaker, allowing a significant fraction of AR27 to still be adsorbed.

Moreover, the presence of a mixture of all previously tested anions (nitrate, chloride, bicarbonate, and sulfate) led to some inhibition of AR27 adsorption, although to a lesser extent than sulfate and bicarbonate individually. This behavior may be related to competitive interactions among the anions in solution and the dynamic equilibrium of adsorption processes on the zeolite surface.

The results obtained are consistent with the literature, which indicates that the interference of anions in dye adsorption depends on factors such as valence (Dong et al. 2018), hydration state, and chemical structure of the ions (Li and Liu 2025). Previous studies have highlighted that polyvalent anions, such as sulfate, often reduce the adsorption efficiency of anionic dyes due to competition for active sites and modification of the adsorbent’s surface charge (Liang et al. 2023).

#### Mechanism of adsorption and multilayer formation

The adsorption of the AR27 dye onto the zeolite surface likely occurs through a synergistic mechanism, primarily involving electrostatic interactions favored by the extremely low pH conditions. In an acidic medium (pH 2), the zeolite acquires a positive charge, which promotes strong attraction toward the sulfonate groups (–SO_3_^−^) present in the AR27 structure. These groups, derived from sulfonic acid, remain predominantly dissociated even under acidic conditions, as sulfonic acid is a strong acid and its anion (SO_3_^−^) is highly stable and resistant to protonation (Previdello et al. 2006; Lim et al. 2021). Thus, the ionic interaction between the positively charged surface of the zeolite and the dye’s sulfonate groups plays a crucial role in anchoring the first layer of molecules, constituting the initial step of the adsorption process.

Evidence for this electrostatic interaction is further supported by experiments conducted with competitive anions (Cl^−^, SO_4_^2−^, NO_3_^−^, and HCO_3_^−^), which, by competing for the same positively charged regions of the zeolite, resulted in a reduction in AR27 adsorption capacity. This behavior reinforces the hypothesis that electrostatic attraction is one of the main mechanisms in the initial stage of the adsorption process (Hu et al. 2013).

Beyond electrostatic attraction, hydrogen bonding can also occur between the hydroxyl (–OH), sulfonate (–SO_3_^−^), and azo (–N = N–) groups of the dye and the surface oxygen atoms of the zeolite’s aluminosilicate structure (such as those present in silanol (–Si–OH) and aluminol (–Al–OH) groups) (Ahmad and Kumar 2010; Scheufele et al. 2016; Schulman et al. 2020), acting as a complementary interaction. Additionally, ion-π interactions between the positively charged surface of the zeolite and the aromatic system of AR27 may contribute to the stabilization and orientation of dye molecules on the adsorbent (Thomas et al. 2000; Ramamurthy et al. 2003; Mahadevi and Sastry 2013), facilitating the formation of a well-organized monolayer.

In the formation of subsequent multilayers, interactions predominantly occur between the dye molecules themselves and those already adsorbed onto the initial layer. π–π stacking between the aromatic rings of AR27 molecules favors packing and vertical growth of the adsorption layers (Guo et al. 2023; Zhang et al. 2016). This effect is particularly relevant due to the polycyclic structure of the dye (Fig. S11, Section S5 of the supplementary material), which facilitates the parallel alignment of the aromatic systems. Additionally, Van der Waals forces and hydrogen bonding between the sulfonate, azo, and hydroxyl groups of adjacent molecules can stabilize the upper layers, acting synergistically with π–π interactions (Scheufele et al. 2016).

**Fig. 11:**
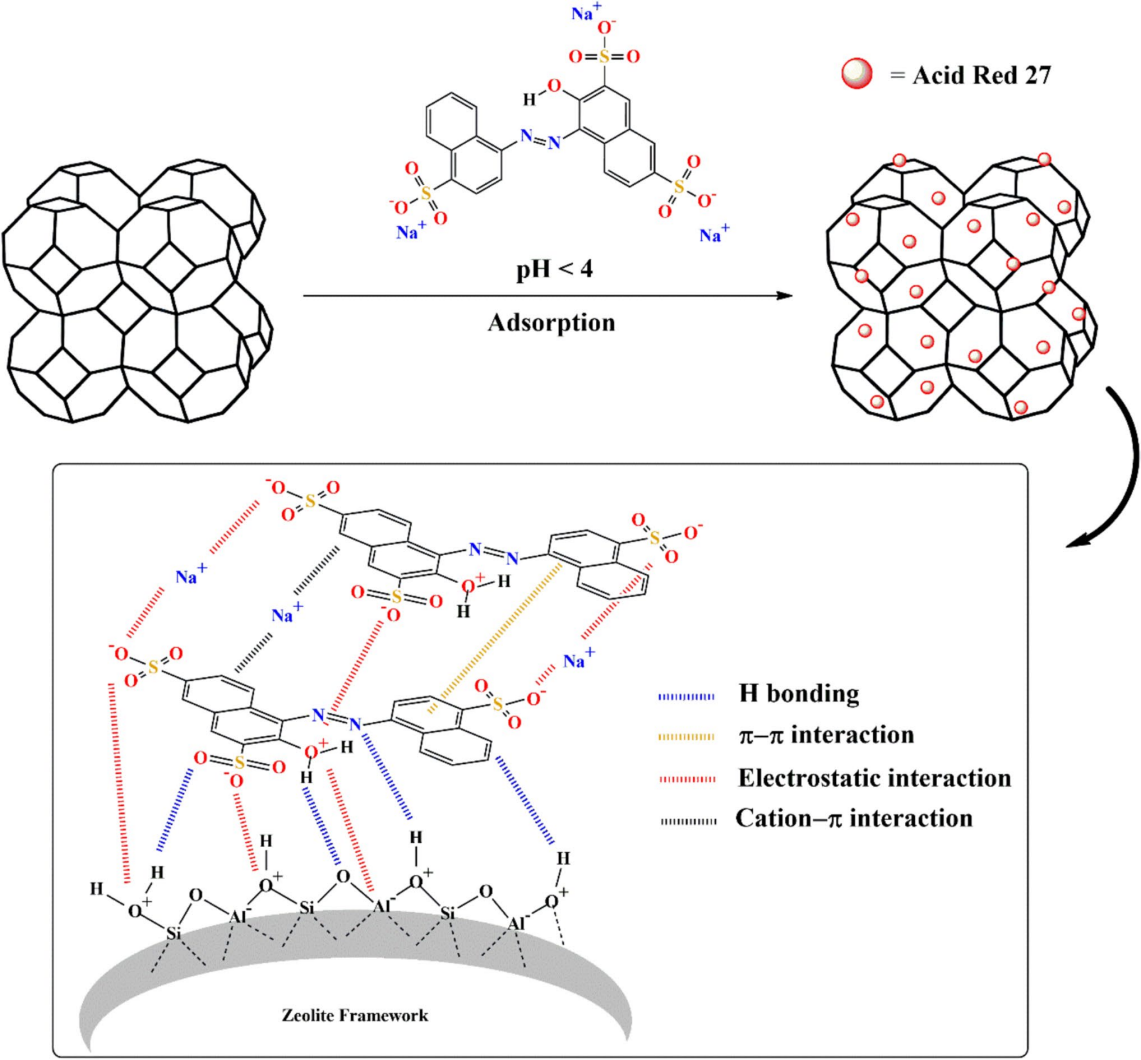
Simplified diagram of the adsorption mechanism of the dye (AR27) on the zeolitic compound

Based on experimental evidence and supported by literature reports (Phan and Kim 2020; Bensaid et al. 2025), a schematic representation of the proposed adsorption mechanism of Acid Red 27 onto the synthesized zeolite is presented in Fig. 11. Overall, the adsorption process can be described as a two-stage mechanism.

In the first stage, dye–surface interactions dominate and are strongly favored under acidic conditions. The pHpzc analysis indicates that the zeolite surface is positively charged at pH values below 7.91, which enhances the adsorption of anionic species such as AR27. In this stage, electrostatic attraction and hydrogen bonding occur mainly between the sulfonate groups of the dye and the hydroxyl (–OH) and oxonium (–OH_2_^+^) groups present on the zeolite surface, promoting the anchoring of the first molecular layer.

In the second stage, multilayer formation becomes predominant and is governed mainly by intermolecular interactions among AR27 molecules already adsorbed on the surface. These interactions include π–π stacking between aromatic rings, as well as electrostatic and cation–π interactions involving exchangeable sodium cations (Na⁺) associated with the zeolite framework and the aromatic system of the dye. The combined contribution of these forces leads to a cooperative and energetically favorable multilayer growth, resulting in a complex adsorption process consistent with the observed equilibrium behavior.

#### Post-adsorption characterization

**Fig. 12:**
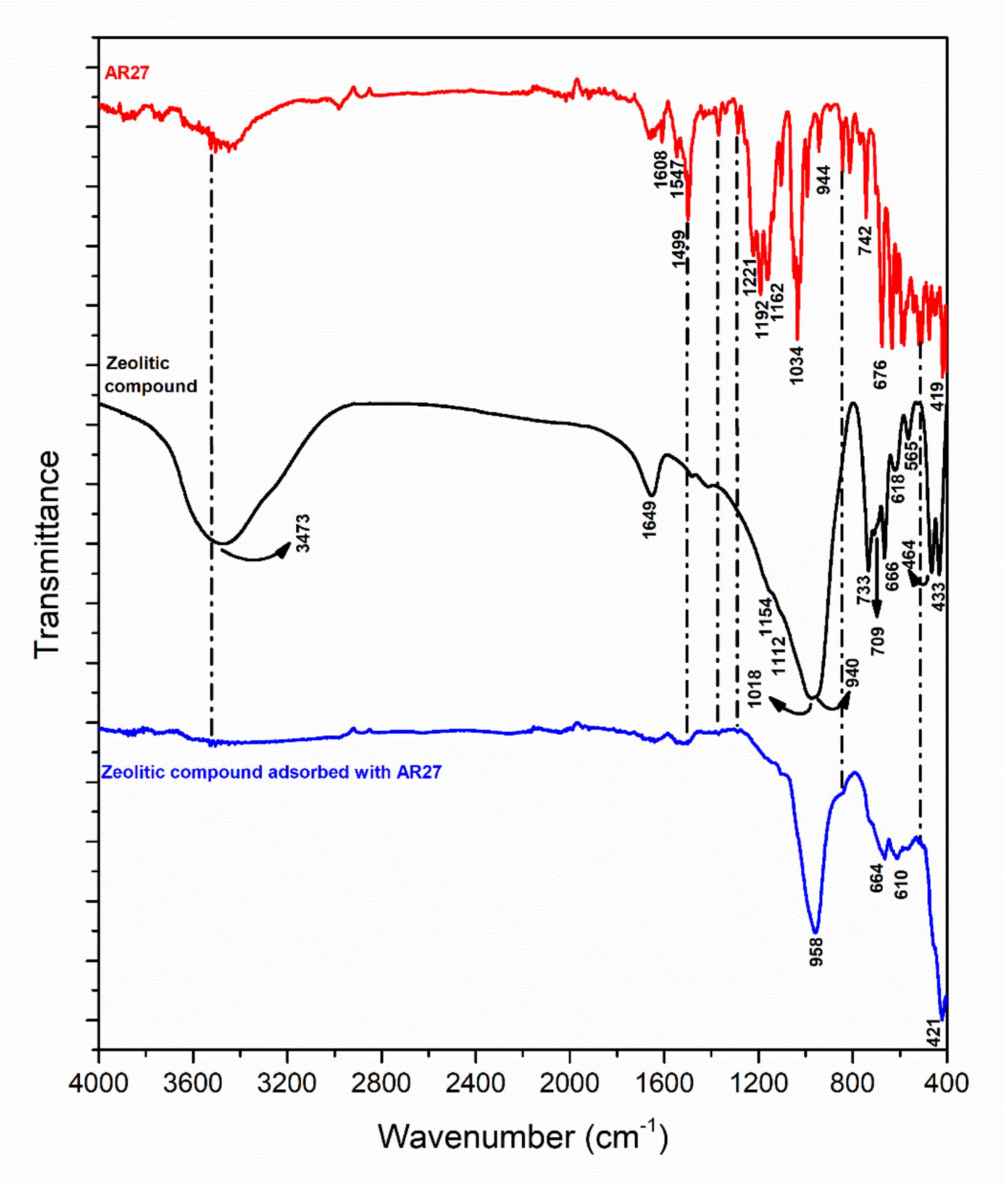
FTIR spectra of the AR27 dye and of the pristine zeolitic compound and after dye adsorption

To investigate the adsorption of the AR27 dye on the surface of the zeolitic compound and to provide evidence for the proposed mechanism, the material saturated after the adsorption process was characterized by FTIR and SEM/EDS. Figure 12 presents the FTIR spectra of the AR27 dye and the synthesized zeolite before and after dye adsorption.

The FTIR spectrum of the AR27 dye exhibits bands characteristic of the functional groups present in its molecular structure. The band around 1499 cm^−1^ stands out, associated with the vibrations of the aromatic ring and/or the azo group (Çatıkkaş et al. 2013; Vinayak and Singh 2022), as well as intense bands in the 1200–1000 cm^−1^ region (1192 and 1034 cm^−1^), typical of stretching vibrations of sulfonate groups (–SO_3_^−^) (Vinayak and Singh 2022; Sismanoglu and Buran 2025) and C–N bonds (Silverstein et al. 2005; Çatıkkaş et al. 2013). Additional bands below 1000 cm^−1^, such as those observed at approximately 944 and 676 cm^−1^, are attributed to out-of-plane deformations of the aromatic rings, composing the vibrational fingerprint of the dye (Ramírez-Hernández et al. 2019; Asemani and Rabbani 2020).

As discussed in Section “Infrared spectroscopy analysis”, the FTIR spectrum of the pure zeolite is characteristic of aluminosilicate materials, being dominated by vibrations of the TO_4_ framework (T = Si or Al), in addition to contributions associated with zeolitic water and surface hydroxyl groups. In this context, the zeolite spectrum is used as a structural reference for evaluating the spectral modifications induced by dye adsorption.

After AR27 adsorption, the spectrum of the dye-loaded zeolite shows evident changes compared to the pristine material, indicating interaction between the adsorbent and the adsorbate. Changes are observed in the intensity and profile of the broad O–H band, suggesting the involvement of zeolite hydroxyl groups and water molecules in the formation of hydrogen bonds with the functional groups of the dye (Huaman et al. 2024). Additionally, the region associated with T–O–T vibrations (950–1250 cm^−1^ and 650–750 cm^−1^) shows variations in intensity and slight spectral reorganization, indicating that the presence of the dye modifies the local chemical environment of the zeolitic framework, possibly through electrostatic interactions between the sulfonate groups of AR27 and the positively charged surface of the zeolite.

In the spectrum of the adsorbed material, bands are also observed in the region below 1000 cm^−1^, such as those around 958, 660, and 610 cm^−1^, which are attributed to the overlap of vibrational modes of the dye with the framework vibrations of the zeolite. The reduction of some characteristic bands of AR27 is attributed to the restriction of vibrational mobility of the dye molecules when adsorbed on the surface or confined within the pores of the material. Similar behavior has been reported in studies on the adsorption of dyes onto zeolites and other porous adsorbents, in which shifts, band broadening, variations in intensity of some characteristic dye bands in FTIR spectra are interpreted as direct evidence of effective adsorption (Çalışkan et al. 2019; Von-Kiti et al. 2023).

Thus, the changes observed in the FTIR spectrum of the zeolite after adsorption, together with the appearance of contributions attributed to AR27 and the modifications in the framework and O–H group bands, confirm the effective retention of the dye. These results indicate that the adsorption process occurs predominantly through electrostatic interactions and hydrogen bonding, without compromising the crystalline structure of the material.

**Fig. 13:**
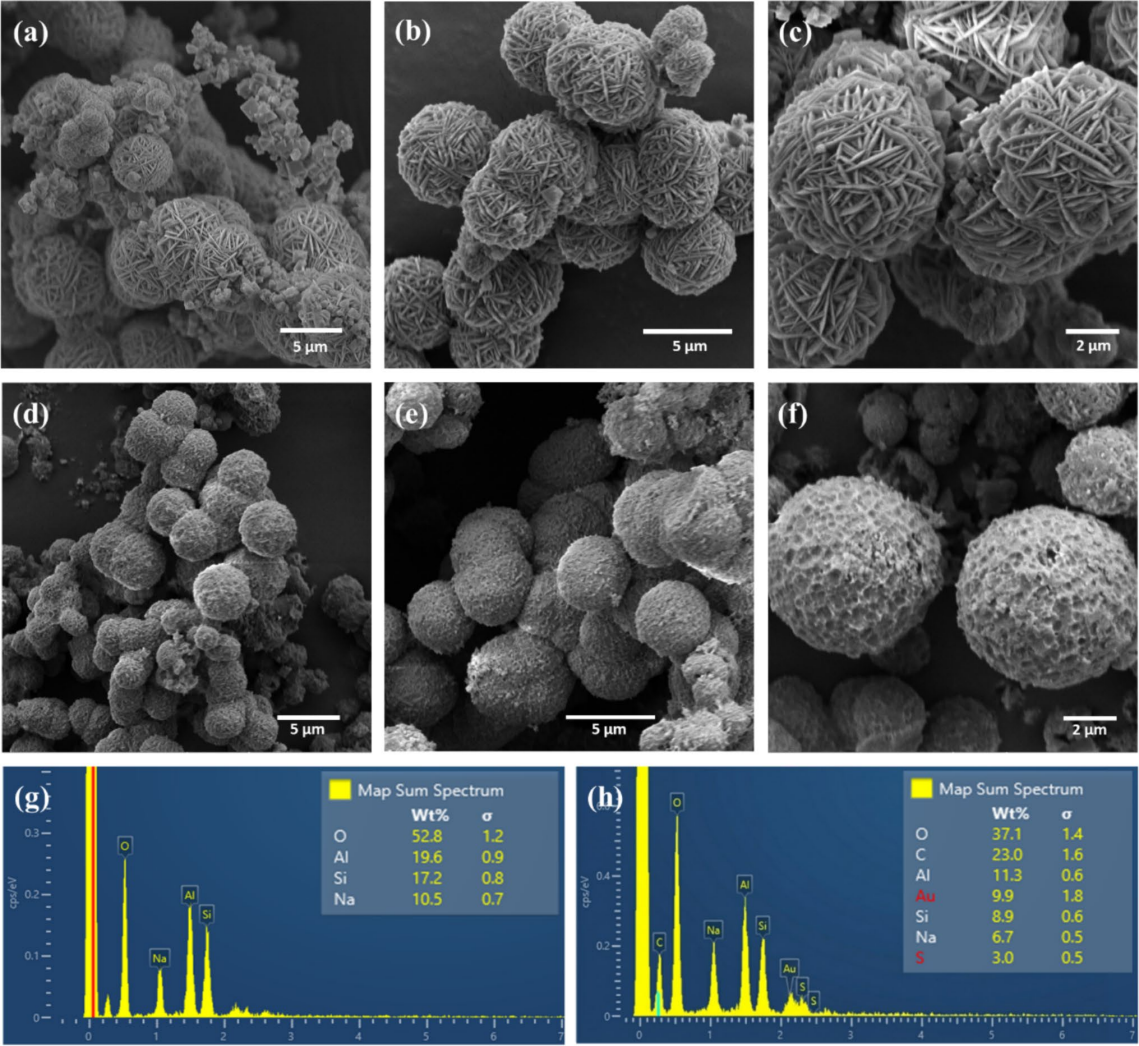
SEM and EDS analyses of the zeolitic compound: **a–c** pristine material (before adsorption); **d–f** material saturated with AR27 dye (post-adsorption); **g, h** EDS spectra of the pristine material and the AR27-loaded material, respectively

SEM images of the pristine material (Fig. 13a–c) were previously discussed in Section “Scanning electron microscopy”. In contrast, micrographs of the dye-saturated material (Fig. 13d–f) reveal significant surface modifications, characterized by the formation of a continuous layer over the crystals, attributed to an organic film resulting from the coverage of external active sites. This surface modification constitutes additional visual evidence of the presence of organic matter adhered to the adsorbent after adsorption.

EDS analysis provides direct elemental evidence of adsorption, based on the detection of sulfur (S), a characteristic element of the dye. The spectrum of the pristine zeolitic material (Fig. 13g) confirms its aluminosilicate nature, showing peaks of silicon (Si), aluminum (Al), sodium (Na), and oxygen (O), with no detectable sulfur. After adsorption (Fig. 13h), the appearance of a sulfur peak is observed. Considering that AR27 contains sulfonate groups as its main anionic functions (Fig. S11, Section S5 of the supplementary material), the presence of sulfur on the material surface confirms the retention of the dye molecules. Gold (Au) was also detected because of the Au layer sputtered during sample preparation in order to make the sample conductive.

Thus, the FTIR, SEM, and EDS analyses complement each other, providing consistent spectroscopic, morphological, and elemental evidence of the effective adsorption of the AR27 dye by the zeolitic compound.

#### Regeneration and reuse of zeolitic compound

**Fig. 14:**
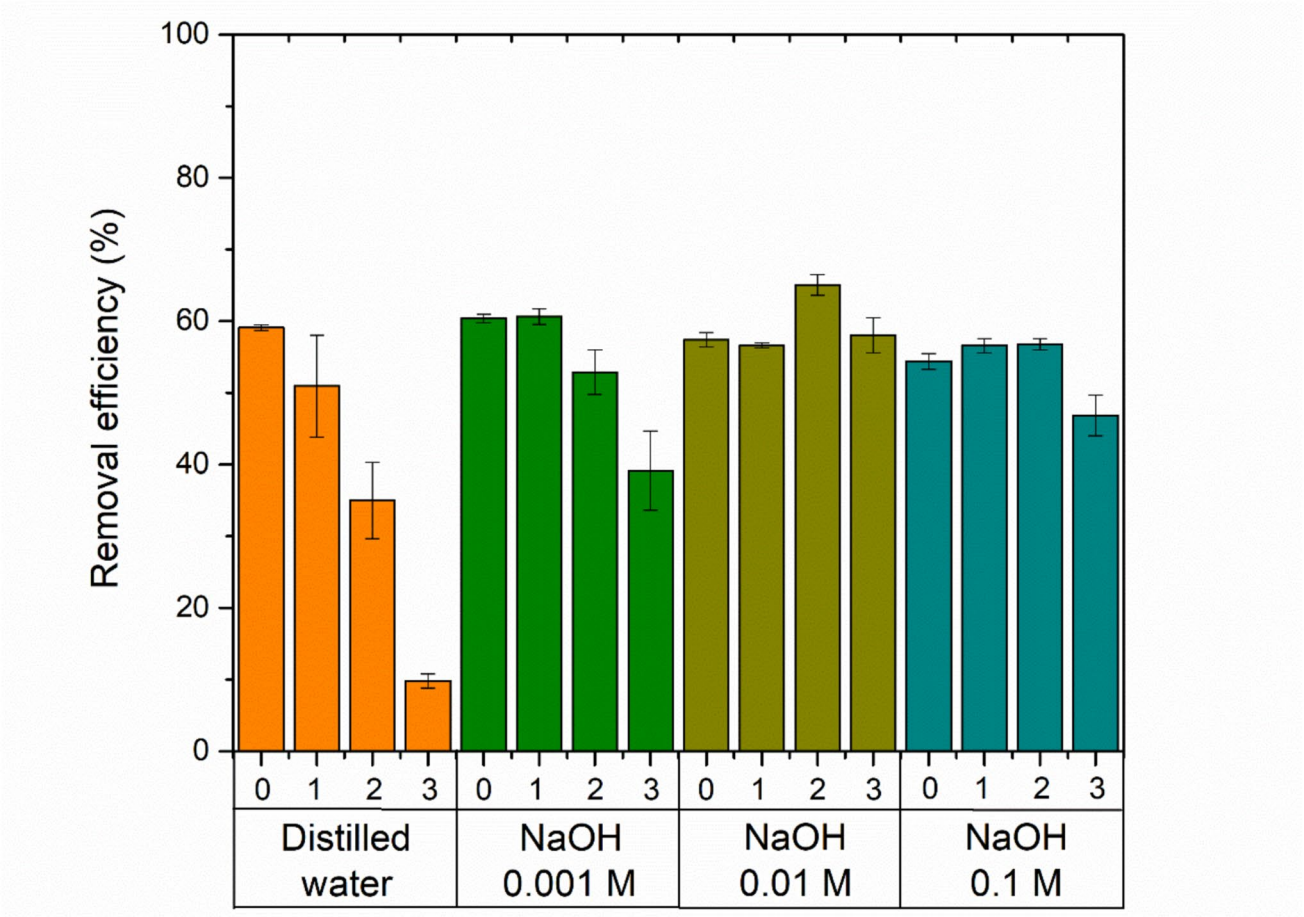
Cyclic batch tests of adsorption and desorption after NaOH regeneration (conditions applied: 90 mg·L^−1^ of initial dye concentration at pH_0_ 2, 1.5 g·L^−1^ of adsorbent, agitation at 300 rpm, temperature fixed at 25 ± 1 °C, and contact time maintained until equilibrium was achieved)

The regeneration of the zeolite as an adsorbent was evaluated using different eluents, including distilled water and NaOH solutions at concentrations of 0.001 M, 0.01 M, and 0.1 M. The experiment was conducted over three consecutive cycles (Fig. 14).

The results show that regeneration with distilled water leads to a sharp decline in removal efficiency over the cycles, dropping from around 60% in cycle 0 (first adsorption) to below 40% in cycle 2, and under 20% in cycle 3. When 0.001 M NaOH is used, the removal efficiency is maintained in the first cycle but progressively decreases in subsequent cycles, reaching approximately 40% by cycle 3. In contrast, regeneration with 0.01 M NaOH demonstrates greater stability, with a slight increase in efficiency in cycle 2, surpassing 70%, and maintaining values above 60% through cycle 3. The 0.1 M NaOH solution also performs well in the first two cycles, with efficiency above 60%, but shows a notable decline in the third cycle, falling below 50%. These results suggest that 0.01 M NaOH provides the most stable and effective regeneration across the evaluated cycles.

Variations in adsorption performance over multiple regeneration cycles may be attributed to changes in the zeolite’s porosity, morphology, and possibly crystalline characteristics (Ates 2018). Zeolites are sensitive to alkaline environments, which can induce increased porosity through the partial dissolution of silica and alumina components. Silica is primarily removed by cleavage of siloxane bonds and subsequent silicon leaching (a process known as desilication), while dealumination tends to be less pronounced, as Si–O–Si bonds are more susceptible to alkaline attack than Si–O–Al bonds (Garcia-Basabe et al. 2010; Ates and Akgül 2016). These processes are influenced by both the concentration of the alkaline solution and the chemical composition of the zeolite (Ates 2018; Jin Et al. 2021), and they can enhance the diffusion of larger molecules into the adsorbent matrix. However, excessively high alkaline concentrations may cause severe structural damage, reducing the material’s adsorptive capacity (Ates 2018). The maintenance of the removal efficiency observed with 0.01 M NaOH may be related to its ability to promote dye desorption, associated with a slight alteration in the zeolite’s surface texture without significantly compromising its crystalline structure and adsorptive properties.

**Fig. 15:**
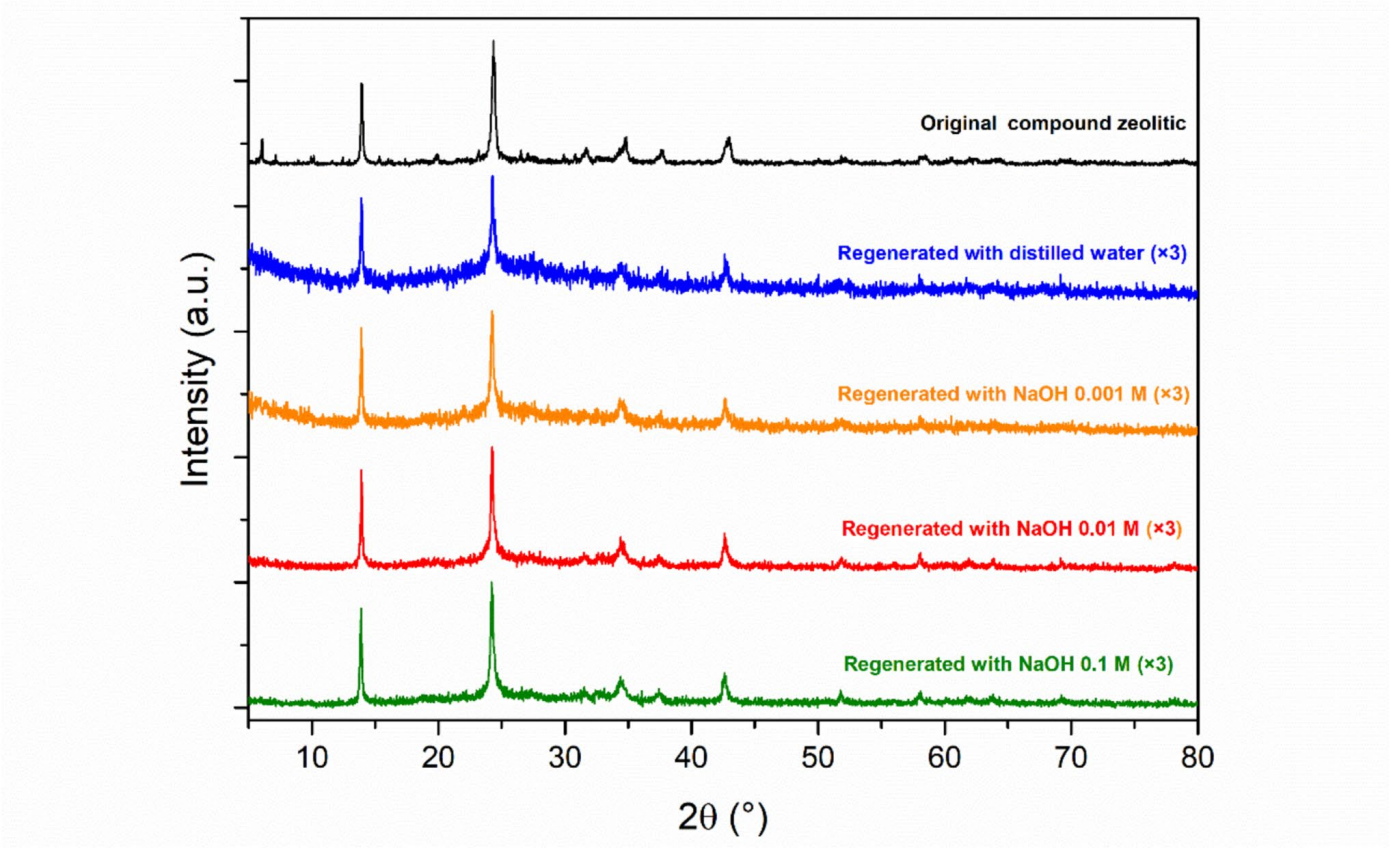
XRD patterns of the zeolite after three adsorption–regeneration cycles using distilled water and NaOH solutions at different concentrations (0.001 M, 0.01 M, and 0.1 M)

At low concentrations (e.g., 0.001 M), the NaOH solution lacks sufficient alkalinity to effectively desorb the dye from the zeolite surface, thereby limiting its regeneration potential. Conversely, while higher concentrations such as 0.1 M initially enhance regeneration, they may also induce undesirable surface alterations that affect performance over repeated cycles. XRD patterns of the zeolite after three adsorption and regeneration cycles (Fig. 15) show that the main diffraction peaks remain well-defined even after washing with 0.1 M NaOH. This suggests that the alkaline concentration used in this study was not sufficient to significantly degrade the crystalline structure of the zeolite. This observation aligns with literature reports indicating that NaOH concentrations above 1 M can cause substantial structural damage in natural zeolites (Jeong et al. 2001; Ates 2018), which far exceeds the maximum concentration applied in this research.

**Fig. 16:**
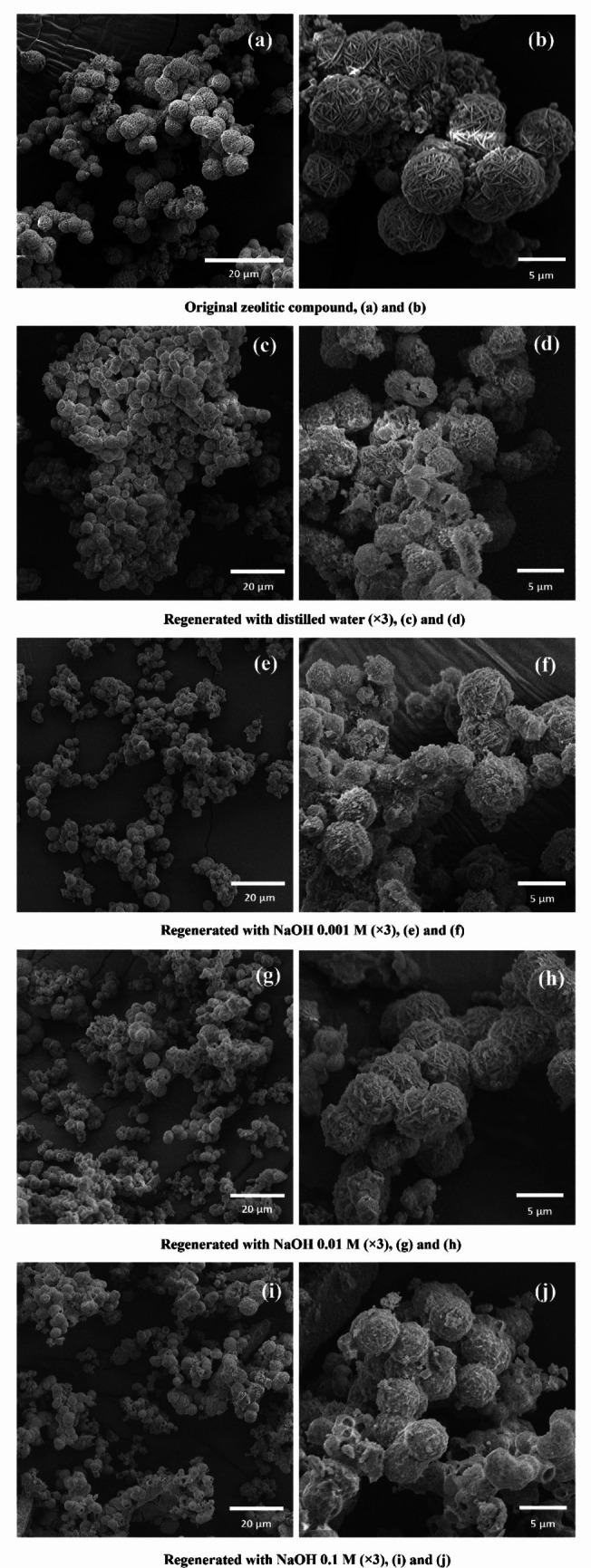
SEM images of the original zeolite and after three regeneration cycles using distilled water, 0.001 M, 0.01 M, and 0.1 M NaOH

However, SEM images (Fig. 16) revealed progressive changes in morphology with increasing NaOH concentration. Compared to the original synthesized zeolite, which exhibited well-defined spherical particles with a texture resembling a ball of yarn formed by intergrown needle-like crystals, the alkali-treated samples showed more compact and uniform spherical structures. Despite this smoother overall appearance, localized surface irregularities and discontinuities became more evident at higher NaOH concentrations, suggesting partial desilication/dealumination. These morphological modifications support the hypothesis that the improvement in regeneration performance, particularly at 0.01 M, may be related to changes in surface texture and enhanced accessibility to active sites.

#### Comparative performance of commercial adsorbents

To evaluate the practical potential of the synthesized zeolite, its adsorption performance was compared to three reference adsorbents: two widely used commercial materials — granular activated carbon (GAC), powdered activated carbon (PAC), and sugarcane bagasse ash (SCBA) used in its raw form (Table 6). Activated carbons are well established in water and wastewater treatment due to their high surface area and broad adsorption profile, while raw SCBA represents a low-cost residue with potential for direct application. The comparison considered isotherm modeling (Langmuir and Freundlich) and empirical constants, aiming to position the synthesized material among practical alternatives for adsorption processes.

**Table 6:** Isotherm model parameters for AR27 adsorption onto the synthesized zeolite, GAC, PAC, and raw SCBA (conditions applied: initial dye concentrations varying between 5 and 1000 mg·L^−1^ at pH_0_ = 2, adsorbent dosages of 1.5 g·L^−1^ (zeolite), 2.8 g·L^−1^ (GAC), 2.5 g·L^−1^ (PAC) and 4.0 g·L^−1^ (SCBA), agitation at 300 rpm, temperature fixed at 25 ± 1 °C (30 ± 1 °C for SCBA), and contact time maintained until equilibrium was achieved)

Parameter	Synthesized zeolite	Granular activated carbon (GAC)	Powdered activated carbon (PAC)	SCBA (Ref: de Santana et al. 2024)
Best fitting isotherm model	BET	Freundlich	Freundlich	Freundlich
*Langmuir:*				
*q*_*max*_ (mg·g^−1^)	497.1239 ± 66.0441	20.6500 ± 2.3321	22.2582 ± 2.3239	15.0083 ± 0.8738
*K*_*L*_ (L.mg^−1^)	0.0022 ± 0.0005	0.0103 ± 0.0031	0.0286 ± 0.0124	0.03675 ± 0.0080
*Freundlich:*				
*K*_*F*_ ([(mg·g^−1^)·(mg·L^−1^)^−1/*n*^])	4.3125 ± 1.5089	1.8970 ± 0.2652	4.3961 ± 0.5030	2.4595 ± 0.2216
*n*	1.5114 ± 0.0384	2.7600 ± 0.1854	3.7618 ± 0.3073	3.0029 ± 0.1779

The synthesized zeolite exhibited a markedly higher Langmuir maximum adsorption capacity than all reference adsorbents. This value should be interpreted as a theoretical indicator rather than an absolute measure of performance (Yaffar et al. 2025), since the Langmuir model was not the best fit for any of the materials, implying that *q*_*max*_ reflects an idealized monolayer capacity. In practical terms, experimental isotherms (Fig. S12, Section S5 of the supplementary material) provide a more reliable comparison, revealing that the zeolite consistently achieved superior equilibrium adsorption capacities (*q*_*e*_) across the entire concentration range, especially at medium and high equilibrium concentrations, outperforming the commercial carbons by a significant margin.

The synthesized zeolite was best described by the BET model, consistent with multilayer adsorption behavior. This differs from GAC, PAC, and SCBA, whose adsorption was better represented by the Freundlich model, indicative of heterogeneous surface sites and variable adsorption energies (Nikam and Mandal 2020; Kalam et al. 2021). This pattern aligns with the irregular pore structure and wide pore size distribution typical of activated carbons (Pelekani and Snoeyink 1999; Ruiz et al. 2010; Salim et al. 2021; Abbas et al. 2022; Sabitov et al. 2024) and unprocessed agricultural ashes (Tan et al. 2015).

For the commercial carbons, the slightly higher Freundlich constant (*K*_*F*_) for PAC compared to GAC suggests improved performance at low equilibrium concentrations, likely due to its finer particle size and, consequently, increased external surface area. Raw SCBA, in turn, showed the lowest maximum adsorption capacity and intermediate Freundlich parameters, indicating limited surface area and pore development compared to processed adsorbents. Nevertheless, its low cost and availability make it a potential candidate for applications where moderate removal efficiency is acceptable and economic constraints are critical. None of the three adsorbents, however, approached the overall adsorption capacity observed for the zeolite.

From an application perspective, the synthesized zeolite shows strong potential for processes requiring high contaminant removal, particularly under moderate to high concentration conditions. Its high capacity, structural stability, and regeneration potential position it as a competitive and efficient option for water and wastewater treatment, clearly outperforming both commercial activated carbon and raw SCBA.

## Conclusions

This study successfully demonstrated a more sustainable route for zeolite synthesis by employing sugarcane bagasse ash and water treatment plant sludge as dual and complementary sources of silicon and aluminum. The obtained zeolite, predominantly of the sodalite type, proved to be an efficient adsorbent for the removal of the Acid Red 27 dye.

In terms of performance, the material exhibited a high experimental maximum adsorption capacity (250 mg·g^−1^), consistently outperforming commercial activated carbons, both granular and powdered. This advantage is particularly evident under medium to high contaminant concentrations, highlighting the waste-derived adsorbent as a promising alternative. Detailed analysis indicated that the adsorption process is best described by a pseudo-second-order kinetic model and a multilayer mechanism (BET model), potentially with initial driven by electrostatic interactions and subsequently complemented by cation-π and π–π stacking in the upper layers.

A crucial aspect for economic and environmental feasibility was the excellent reusability of the material: regeneration with a dilute NaOH solution (0.01 M) allowed its performance to be maintained over three consecutive cycles. On the other hand, the presence of competing anions, such as sulfate and bicarbonate, significantly reduced the removal efficiency, which must be considered in real wastewater matrices with high ionic complexity.

In summary, this work validates the potential of waste-derived zeolite as an inherently low-cost alternative relative to precursors, combining high adsorption capacity with good regenerability for wastewater treatment, in alignment with circular economy principles and sustainable resource management. For future studies, the application of the material in real effluents and the evaluation of its performance in continuous-flow systems are recommended to consolidate its viability at an industrial scale.

## Supplementary information

Below is the link to the electronic supplementary material.

ESM 1 (DOCX 2.87 MB)

## Data Availability

The authors declare that the data supporting the findings of this study are available within the paper and its supplementary information files.
