# Supplementary material for: Waste-derived zeolite from sugarcane bagasse ash and water treatment plant sludge for sustainable industrial dye removal
Source: Environ Sci Pollut Res Int. 2026 Feb 4;33(7):2852–85. doi: 10.1007/s11356-026-37455-y (PMC12982224; doi:10.1007/s11356-026-37455-y)
Supplement: Supplementary file 1 — (DOCX 2.87 MB) [file 11356_2026_37455_MOESM1_ESM.docx]

**Waste-Derived Zeolite from Sugarcane Bagasse Ash and WTP Sludge for Sustainable Industrial Dye Removal**

Joana Eliza de Santana^a*^, Antônio Elias dos Santos Neto^a^, Fábio Gabriel Silva de Andrade^a^, Aldebarã Fausto Ferreira^b^, Marcos Gomes Ghislandi^c^, Maurício Alves da Motta Sobrinho^a^

^a^Chemical Engineering Department, Federal University of Pernambuco, Recife, PE, 50.740-590, Brazil.

^b^Departament of Fundamental Chemistry, Federal University of Pernambuco, Recife, PE, 50.740-590, Brazil.

^c^Engineering Campus, Federal Rural University of Pernambuco, Cabo de Santo Agostinho, PE, 54518-430, Brazil.

*Corresponding author: [joana.santana@ufpe.br](mailto:joana.santana@ufpe.br)

**SUPPLEMENTARY INFORMATION**

**S1. Adsorption Models: Kinetic and Isotherm Equations**

**Table S1**

Mathematical expressions of the kinetic models used for Acid Red 27 adsorption analysis.

| *Kinetic model* | *Expression* | *Equation No.* | *Reference* |
| --- | --- | --- | --- |
| Pseudo-first-order | $\text{q}_{\text{t}}\text{ = }\text{q}_{\text{e}}\text{(1- }\text{e}^{\text{-}\text{k}_{\text{1}}\text{t}}\text{)}$ | (S1) | Bujdák 2020 |
| Pseudo-second-order | $\text{q}_{\text{t}}\text{= }\frac{{{\text{k}_{\text{2}}\text{q}}_{\text{e}}}^{\text{2}}\text{t}}{\text{1+ }\text{k}_{\text{2}}\text{q}_{\text{e}}\text{t}}$ | (S2) | Ho and McKay,1999; Bujdák 2020 |
| Pseudo-n-order | $\text{q}_{\text{t}}\text{= }\text{q}_{\text{e}} \text{- }\frac{\text{q}_{\text{e}}}{{\text{[1 + }\text{k}_{\text{n}}{\text{(}\text{q}_{\text{e}}\text{)}}^{\text{n-1}}\text{t(n-1)]}}^{\frac{\text{1}}{\text{n-1}}}}$ | (S3) | Peres et al. 2018 |
| Intraparticle diffusion (Weber-Morris) | $\text{q}_{\text{t}}\text{ = }\text{k}_{\text{id}}\text{ }\text{t}^{\text{0.5}}\text{+C}$ | (S4) | Hubbe et al. 2019 |

In these equations, t denotes the contact time (min); qₜ and qₑ represent the adsorptive capacity at time t and at equilibrium (mg·g⁻¹), respectively. The parameter k₁ is the pseudo-first-order rate constant (min⁻¹), k₂ is the pseudo-second-order rate constant (g·mg⁻¹·min⁻¹), while kₙ is the rate constant associated with the pseudo-n-order model (min⁻¹(g·mg⁻¹)ⁿ⁻¹), n corresponds to the reaction order, k_id_ is the rate constant for intraparticle diffusion, and C is a constant associated with the boundary layer thickness (i.e. the “film” for the external film diffusion model).

**Table S2**

Mathematical formulations of adsorption isotherm models considered in this study.

| *Isotherm model* | *Expression* | *Equation No.* | *Reference* |
| --- | --- | --- | --- |
| Langmuir | $\text{q}_{\text{e}}\text{ = }\frac{{\text{q}_{\text{max}}\text{k}}_{\text{L}}\text{C}_{\text{e}}}{\text{1+ }\text{k}_{\text{L}}\text{C}_{\text{e}}}$ | (S5) | Lagmuir 1918; Tran et al. 2021 |
| Freundlich | $\text{q}_{\text{e}}\text{= }\text{K}_{\text{F}}{\text{C}_{\text{e}}}^{\text{1/n}}$ | (S6) | Freundlich, 1906; Tran et al. 2021 |
| BET | $\text{q = }\text{q}_{\text{m}}\left[ \frac{\text{K}_{\text{S}}\text{C}_{\text{e}}\text{[1-(n+1)}{\text{(}\text{K}_{\text{S}}\text{C}_{\text{e}}\text{)}}^{\text{n}}\text{+n}{\text{(}\text{K}_{\text{L}}\text{C}_{\text{e}}\text{)}}^{\text{n+1}}\text{]}}{\text{(1-}\text{K}_{\text{L}}\text{C}_{\text{e}}\text{)[1+(}\frac{\text{K}_{\text{S}}}{\text{K}_{\text{L}}}\text{-1)}\text{K}_{\text{L}}\text{C}_{\text{e}}\text{-(}\frac{\text{K}_{\text{S}}}{\text{K}_{\text{L}}}\text{)}{\text{(}\text{K}_{\text{L}}\text{C}_{\text{e}}\text{)}}^{\text{n+1}}} \right]$ | (S7) | Ebadi et al. 2009 |
| Dubinin–Radushkevich | $\text{q}_{\text{e}}\text{= }\text{q}_{\text{DR}}\text{e}^{\text{-}\text{K}_{\text{DR}}\text{ε}^{\text{2}}}$  $\text{ε = RT ln}\left( \text{1 + }\frac{\text{1}}{\text{C}_{\text{e}}} \right)\text{ }$  $\text{E}_{\text{DR}}\text{ = }\left( \text{2 }\text{K}_{\text{DR}} \right)^{\text{-0.5}}$ | (S8) | Şenol et al. 2020 |

In the above equations, qₑ is the adsorptive capacity at equilibrium (mg·g⁻¹), Cₑ is the concentration of the dye in solution at equilibrium (mg·L⁻¹), qₘₐₓ is the maximum adsorption capacity of the monolayer (mg·g⁻¹), K_L_ is the Langmuir constant (L·mg⁻¹), K_F_ represents the Freundlich constant associated with the adsorption capacity of the material [(mg·g^-1^).(mg·L^-1^)^-1/n^], n (Freundlich model) indicate the adsorption intensity, qₘ is the monolayer adsorption capacity (mg·g⁻¹), K_S_ is the equilibrium constant of adsorption for the first layer (L·mg⁻¹), K_L_ is the quilibrium constant of adsorption for upper layers (L·mg⁻¹), n (BET model) represents the number of layers, K_DR_ is the constant related to the sorption energy (mol^2^·J⁻^2^), q_DR_ is the adsorption capacity (mg·g⁻¹), ε is the Polanyi potential (J·mol^-1^), R is the universal gas constant (8.314 J·mol^-1^·K^-1^), T is the absolute temperature (K), and E_DR_ is the mean free energy of adsorption (kJ·mol^-1^) and represents the energy required to transfer one mole of adsorbate from the bulk solution to the surface of the solid.

**S2. Characterization of sugarcane bagasse ash and WTP sludge**

The ashes studied in this section were kindly provided by sugarcane mills located in Lagoa de Itaenga (Mill 1), Igarassu (Mill 2) and Vitória de Santo Antão (Mill 3), all in the state of Pernambuco, Brazil. The sludges were provided by the Companhia Pernambucana de Saneamento (COMPESA) and collected in dehydrated form from two water treatment plants: Agrestina Nova (WTP 1) in Agrestina, PE, Brazil, and Gurjaú (WTP 2) in Cabo de Santo Agostinho, PE, Brazil.

Fig. S1 shows the X-ray diffraction (XRD) patterns of ashes samples obtained from different sugarcane mills, as well as from different harvests of the same mill. Crystalline quartz (SiO₂) was detected in all samples, in accordance with the reference pattern (COD 96-901-1494), confirming its presence in the residues. This information is supported by the X-ray fluorescence (XRF) results presented in Table S3, which show high silicon contents in all samples, ranging from 183,010 to 317,000 mg·kg⁻¹. Aluminum is also noteworthy, with significant concentrations ranging from 31,000 to 64,000 mg·kg⁻¹.


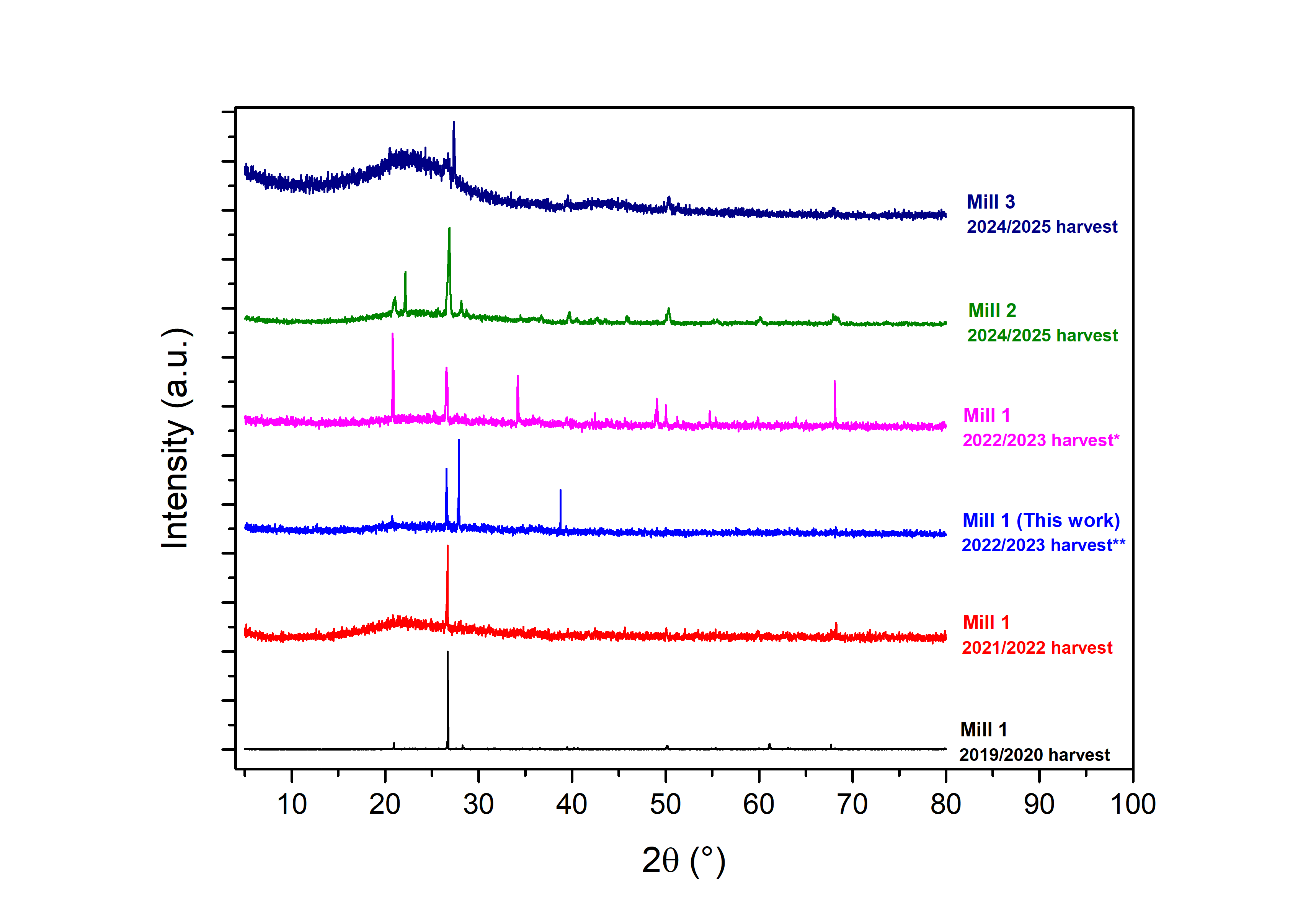


**Fig. S1.** XRD profile of sugarcane bagasse ash from different sugar mills and harvests. (*Ash collected from the upper part of the settling pond; **ash collected from the bottom of the settling pond.)

**Table S3**

Chemical composition (mg·kg⁻¹) of sugarcane bagasse ashes (SCBA) from different sugar mills and harvests, determined by XRF.

| **Elements** | **Mill 1 – 2019/2020 harvest**  **(mg kg^-1^)** | **SD** | **Mill 1 – 2021/2022 harvest**  **(mg kg^-1^)** | **SD** | **Mill 1 – 2022/2023 harvest****  **(mg kg^-1^)** | **SD** | **Mill 1 – 2022/2023 harvest***  **(mg kg^-1^)** | **SD** | **Mill 2 – 2024/2025 harvest**  **(mg kg^-1^)** | **SD** | **Mill 3 – 2024/2025 harvest**  **(mg kg^-1^)** | **SD** |
| --- | --- | --- | --- | --- | --- | --- | --- | --- | --- | --- | --- | --- |
| Si | 317000 | 458 | 246000 | 482 | 183010 | 222 | 237000 | 469 | 242000 | 485 | 208000 | 306 |
| Al | 64000 | 396 | 32000 | 382 | 41755 | 266 | 44000 | 536 | 31000 | 415 | 44000 | 327 |
| Fe | 17000 | 112 | 20000 | 123 | 29317 | 103 | 31000 | 152 | 19000 | 120 | 7599 | 75 |
| Ca | 18000 | 83 | 23000 | 95 | 16880 | 57 | 11000 | 70 | 22000 | 94 | 12000 | 74 |
| Mg | 8044 | 1212 | 14000 | 2478 | 10661 | 1530 | 9348 | 2232 | 14000 | 2516 | 14000 | 2472 |
| K | 37000 | 147 | 26000 | 124 | 15103 | 65 | 26000 | 126 | 28000 | 129 | 19000 | 105 |
| Ti | 1592 | 29 | 1766 | 30 | 3191 | 24 | 3928 | 37 | 2537 | 33 | 1243 | 27 |
| Mn | 872 | 15 | 1033 | 16 | 640 | 9 | 752 | 14 | 877 | 15 | 584 | 15 |
| P | 2784 | 50 | 3294 | 52 | 741 | 21 | 1401 | 38 | 3319 | 52 | 1811 | 42 |
| Zr | 614 | 7 | 227 | 5 | 338 | 3 | 468 | 7 | 544 | 7 | 72 | 5 |
| Zn | 152 | 6 | 314 | 9 | 277 | 7 | 159 | 7 | 346 | 10 | 154 | 7 |
| Cu | 213 | 8 | 190 | 8 | 80 | 5 | 83 | 6 | 87 | 7 | 129 |  |
| Ni | 7 | 6 | 8 | 6 | 16 | 5 | 6 | 6 | <LD |  | <LD |  |
| Cr | <LD |  | <LD |  | 12 | 6 | 52 | 9 | 13 | 9 | <LD |  |
| Pb | 13 | 10 | 22 | 11 | 12 | 5 | 14 | 12 | 14 | 11 | <LD |  |
| LOI | 12.08 | | 23.63 | | 11.16 | | 7.19 | | 14.46 | | 72,87 | |

SD = Standard deviation

LOI = Loss on ignition

LD = Limit of detection

*Ash collected from the upper part of the settling pond

**Ash collected from the bottom of the settling pond

Similarly, Fig. S2 shows the diffraction patterns of sludge samples from two Water Treatment Plants (WTPs). The observed spectra are similar comparing the two samples, suggesting similar mostly amorphous structure, with traces of quartz and kaolinite. Specifically, the sample from the Gurjaú WTP exhibits a slightly higher crystallinity, with more defined peaks, while the sample from the Agrestina Nova WTP shows a more pronounced amorphous halo, indicating a greater amorphous fraction. This characteristic is related to the higher organic matter content in the Agrestina Nova sample, as confirmed by the loss on ignition (LOI) analysis presented in Table S4. The LOI for the Agrestina Nova sample is 58.12%, significantly higher than that of Gurjaú (20.32%), confirming the greater presence of organic components responsible for diffuse diffraction. Despite these differences, the silicon and aluminum contents remain in the same order of magnitude in both samples — with the Agrestina Nova WTP sludge containing 200,000 mg·kg⁻¹ of Si and 100,000 mg·kg⁻¹ of Al, and the Gurjaú WTP sludge containing 161,750 mg·kg⁻¹ of Si and 70,915 mg·kg⁻¹ of Al — which reinforces their potential for zeolite synthesis.


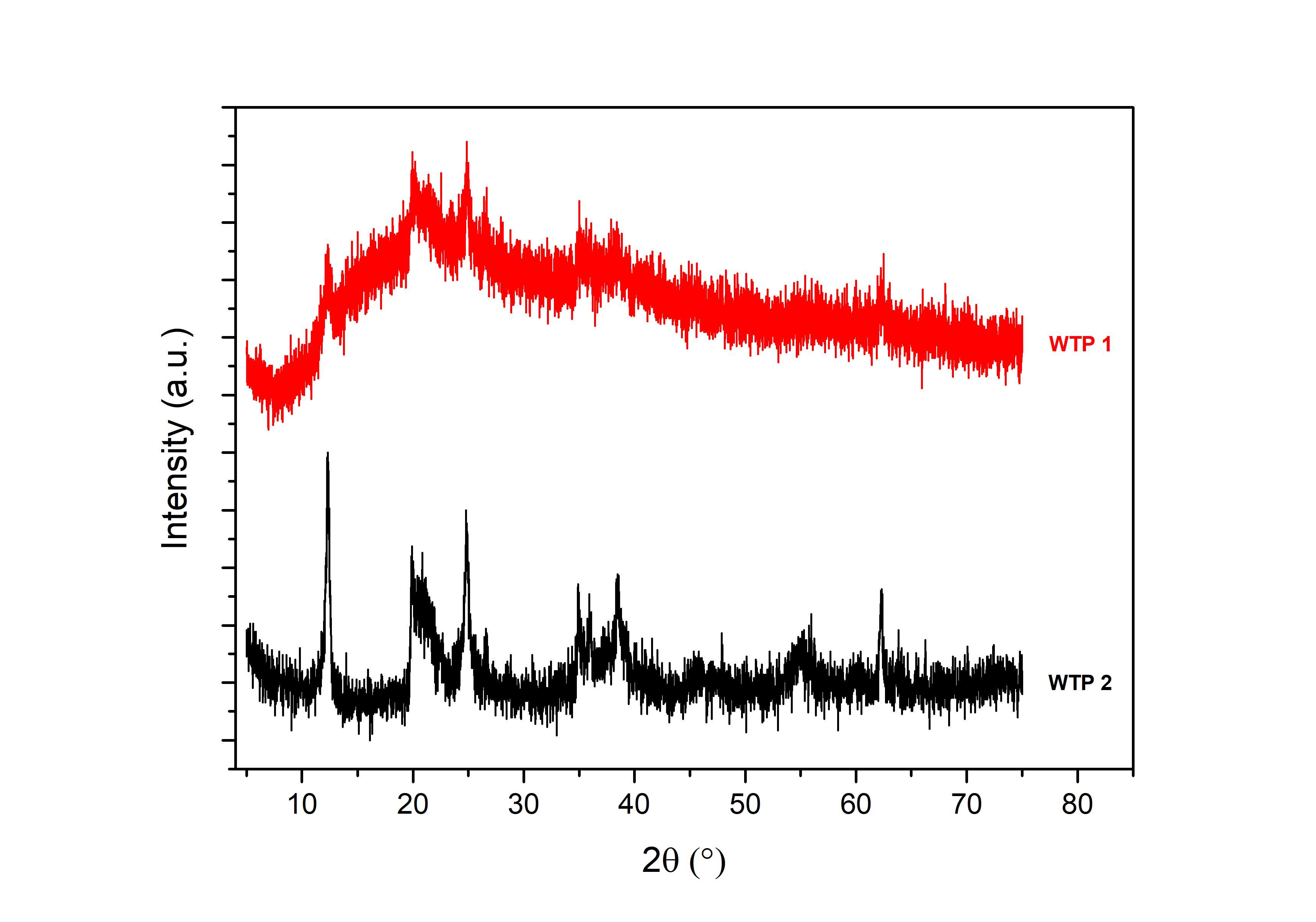


**Fig. S2.** XRD profile of WTP sludge from two different WTPs.

**Table S4**

Chemical composition (mg·kg⁻¹) of of WTP sludge from two different WTPs determined by XRF.

| **Elements** | **WTPS 1**  **(mg kg^-1^)** | **SD** | **WTPS 2**  **(mg kg^-1^)** | **SD** |
| --- | --- | --- | --- | --- |
| Si | 200000 | 187 | 161750 | 212 |
| Al | 100000 | 651 | 70915 | 454 |
| Fe | 95000 | 273 | 49402 | 134 |
| Ca | 3443 | 45 | 622 | 17 |
| Mg | 3704 | 1682 | 4224 | 1109 |
| K | 1859 | 48 | 1841 | 31 |
| Ti | 1269 | 27 | 5556 | 28 |
| Mn | 947 | 17 | 113 | 7 |
| P | 1142 | 41 | 280 | 18 |
| Zr | 16 | 3 | 34 | 2 |
| Zn | 52 | 6 | 61 | 4 |
| Cu | 36 | 7 | 29 | 4 |
| Ni | 8 | 8 | 10 | 5 |
| Cr | 115 | 11 | 99 | 6 |
| Pb | 12 | 7 | 45 | 6 |
| LOI | 58.12% | | 20.32% | |

SD = Standard deviation

LOI = Loss on ignition

LD = Limit of detection

The thermogravimetric analysis (TGA) of sugarcane bagasse ash, presented in Fig. S3, reveals variations in thermal behavior across different harvests and mills. The sample from Mill 3 – 2024/2025 harvest shows the highest total mass loss, with a reduction of about 80% up to approximately 600 °C, indicating a high content of organic matter and volatile compounds. This profile suggests lower combustion efficiency or lower burning temperatures during the transformation of bagasse in the boiler. All sugarcane bagasse ash samples reach thermal stability in the range of approximately 450 to 600 °C, indicating the completion of organic matter degradation. For most samples, total mass losses are below 15%, reflecting a low content of organic matter. Exceptions include the ashes from Mill 3 (2024/2025) and Mill 1 (2021/2022), which show more significant losses, suggesting a higher content of organic or volatile components. The samples from Mill 1 – 2022/2023 harvest, represented by two curves, also display similar thermal profiles, with total mass losses close to and below 15%. The difference between them is less than 5%, indicating minor variations in organic matter content, likely due to different collection points within the settling pond.

The differences observed in the thermal degradation profiles of sugarcane bagasse ash samples are mainly related to the combustion conditions and operating temperatures of the boilers at each plant, which influence both the residual organic matter content and the degree of transformation of the mineral constituents. In general, lower combustion temperatures tend to result in a higher amount of remaining organic compounds, while higher temperatures favor the formation of ashes rich in inorganic compounds, which are more suitable for applications such as zeolite synthesis.

During the analysis of samples from different harvests and plants, it was observed that most had an organic matter content below 20%. However, one specific sample showed an atypical value around 80%, which may suggest the influence of several factors, among which stand out: the moisture content of the biomass at the time of feeding the system, the type and size of the boiler, the bagasse feed rate, as well as combustion efficiency (Payne 2010; Mali and Nanthagopalan 2020). Bagasse with higher moisture content requires more energy to evaporate the water, which can reduce the temperature in the combustion zone and compromise the process efficiency, resulting in a higher amount of organic matter in the ash. In addition, high feed rates can reduce the material's residence time in the boiler, making complete combustion difficult. Smaller boilers or those with inefficient temperature and residence time control are also less effective in the complete thermal degradation of the biomass.


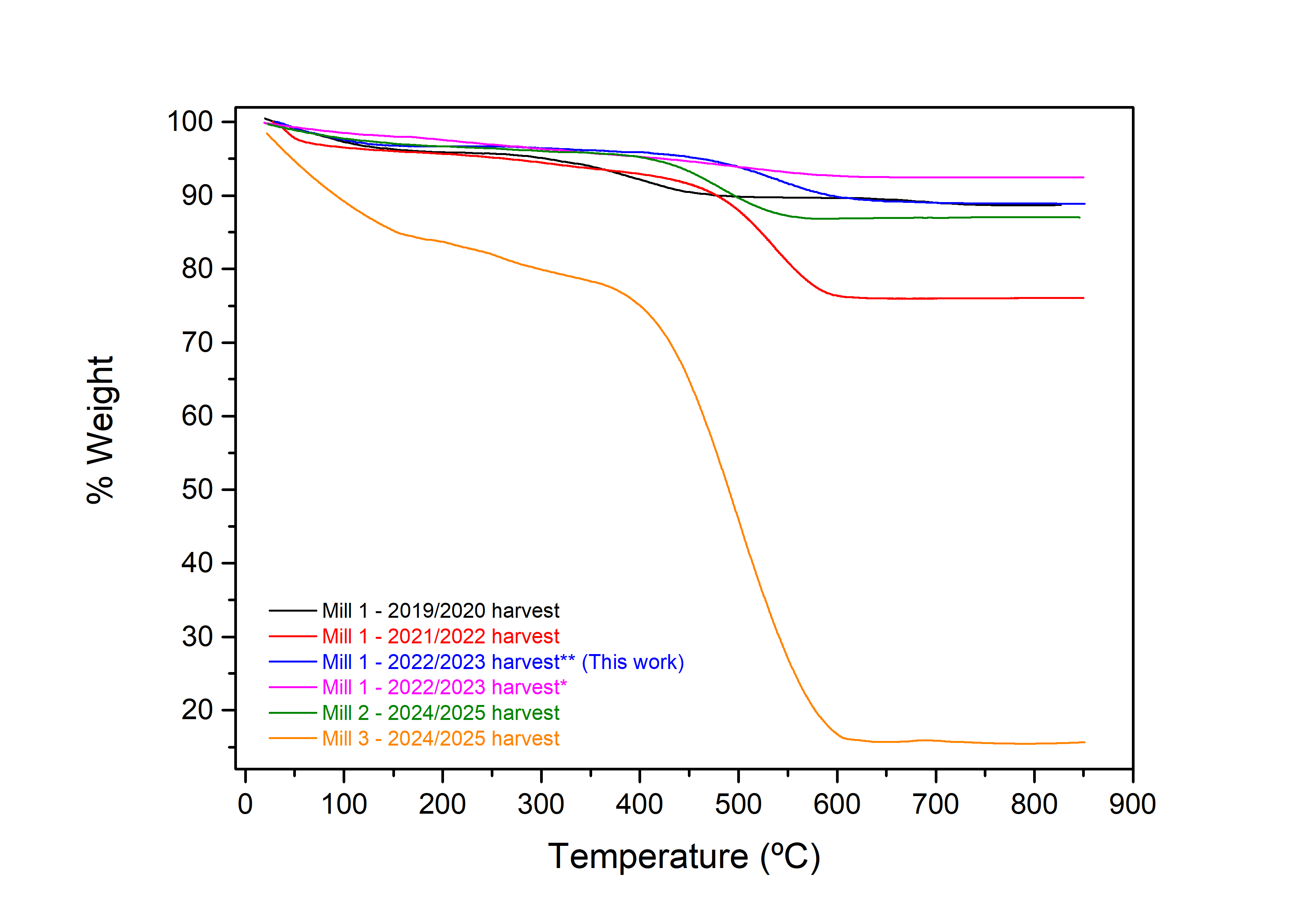


**Fig. S3.** TGA curve of sugarcane bagasse ash from different sugar mills and harvests (*Ash collected from the upper part of the settling pond; **ash collected from the bottom of the settling pond).

In the case of WTP sludge, the TGA results shown in Fig. S4 display the mass losses of the samples. The sample from the Agrestina Nova WTP shows a total mass loss of approximately 60%, while the sample from the Gurjaú WTP shows a loss of around 20%. These values directly reflect the organic matter content in the materials, as indicated by the LOI analysis in Table S4. The higher mass loss observed in Agrestina Nova is therefore associated with its elevated organic content, which may also be related to the greater proportion of amorphous material identified in the XRD pattern.


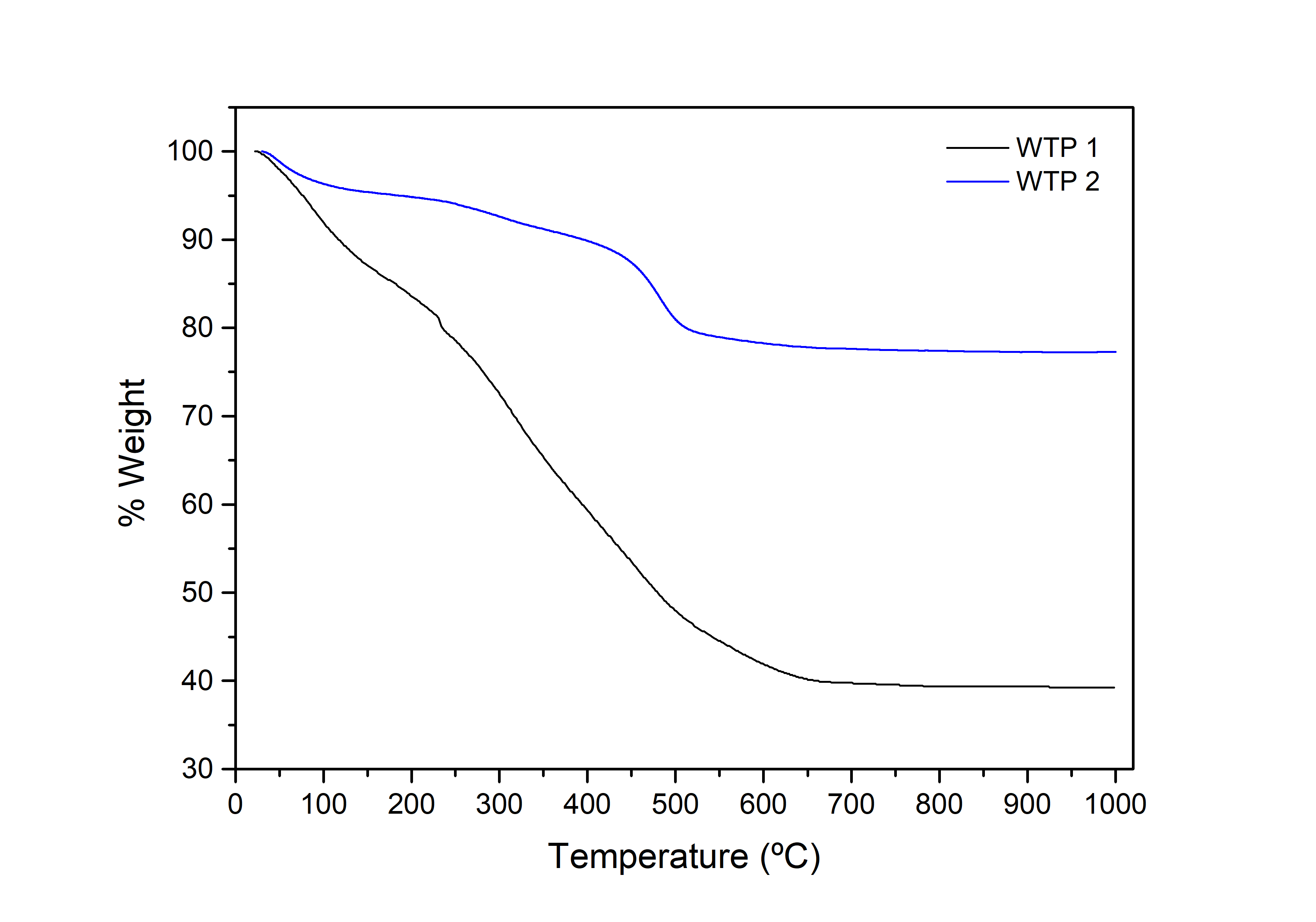


**Fig. S4.** TGA curve of of WTP sludge from two different WTPs.

The Fourier-transform infrared (FTIR) spectra presented in Fig. S5 and S6 highlight the similarities among the analyzed residues. The sugarcane bagasse ash exhibits bands related to Si–O, the symmetric stretching vibration of Si–O–Si in quartz, and the bending vibrations of SiO₄ tetrahedra, indicating compositional consistency over time. Likewise, the spectra of the WTP sludge samples also show similarities, although the sample from the Gurjaú WTP stands out due to more intense and sharper bands, suggesting a higher degree of structural organization and lower organic content. The sample from the Agrestina Nova WTP exhibits bands associated with the presence of hydroxides and metal oxides, evidenced by the stretching of the internal hydroxyl group (Al—O–H) at 3620 cm⁻¹, confirming the presence of kaolinite (Ling et al. 2017). The Si–O bond stretching is also observed, with bands near 1110 cm⁻¹ and 1003 cm⁻¹ (Ling et al. 2017), as well as a planar stretching at 1032 cm⁻¹ (Davarcioglu 2011). A prominent band at 910 cm⁻¹ results from the vibration of the kaolinite sheet, attributed to Al–Al–OH deformation (Ling et al. 2017; Davarcioglu 2011; Saikia and Parthasarathy 2010). Bands at 422 and 463 cm⁻¹ correspond to the bending of the Si–O–Si group (Davarcioglu 2011; Saikia and Parthasarathy 2010). These results corroborate the previously mentioned characterization data.


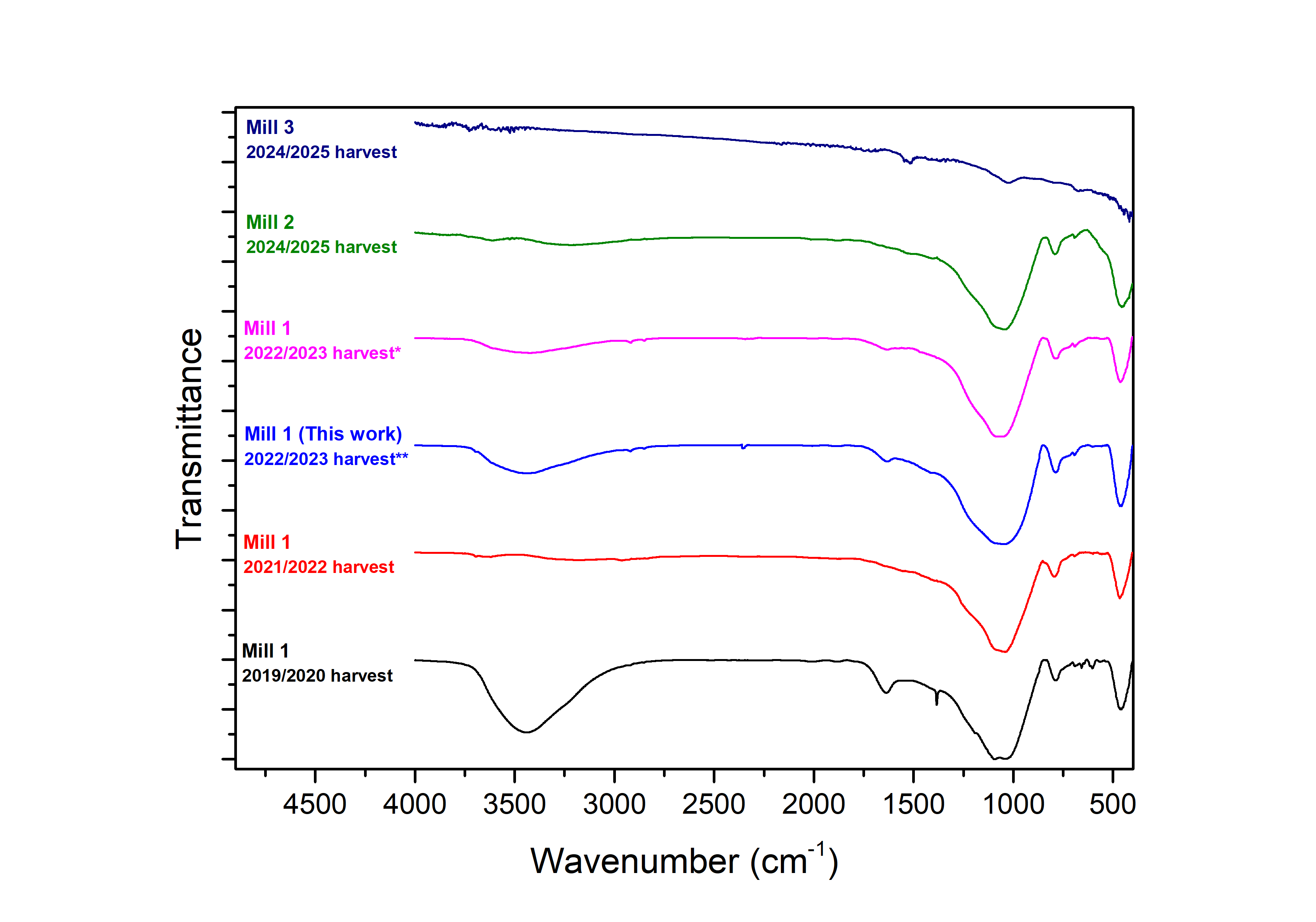


**Fig. S5** Infrared spectra of sugarcane bagasse ash from different sugar mills and harvests (*Ash collected from the upper part of the settling pond; **ash collected from the bottom of the settling pond).


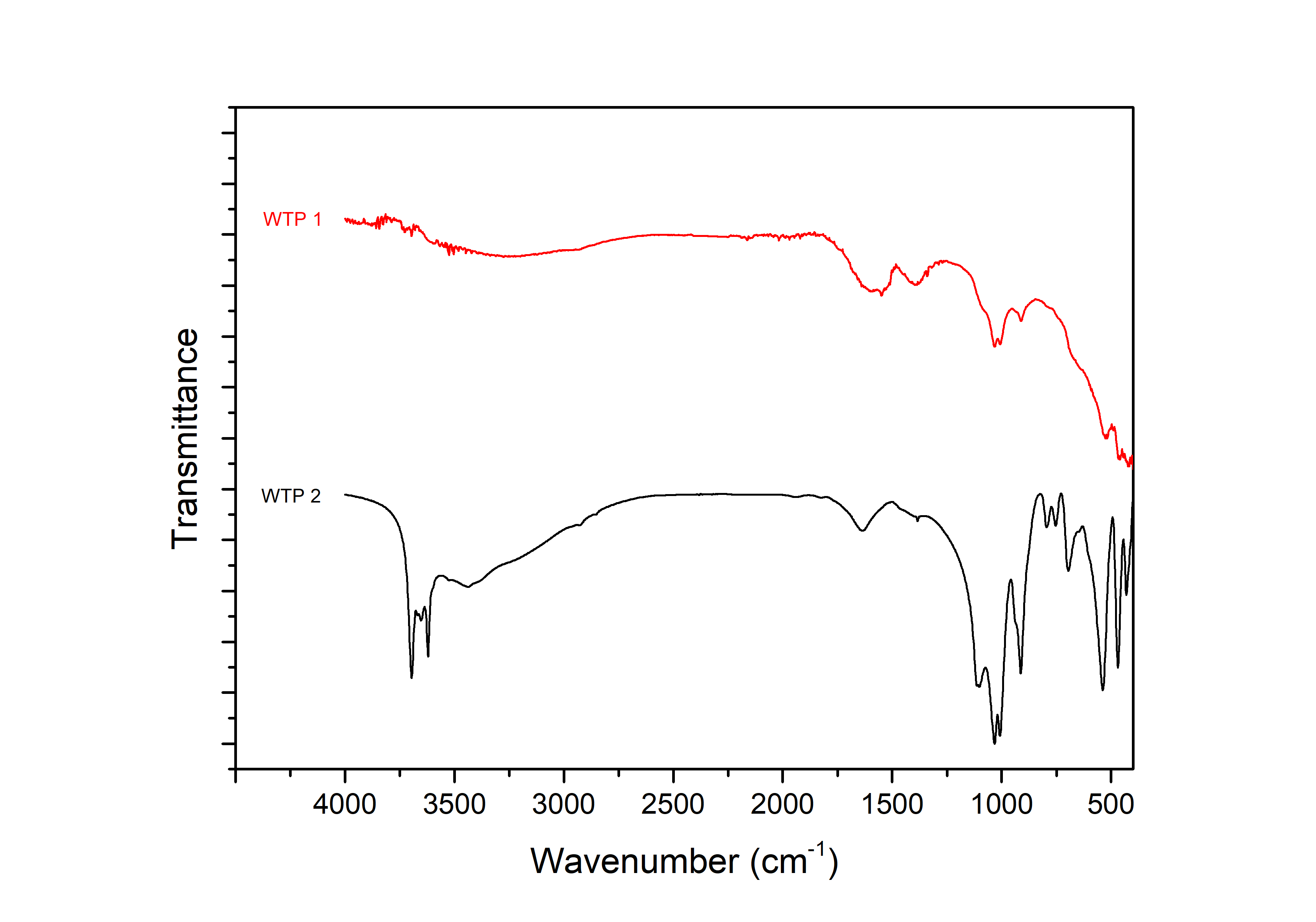


**Fig. S6.** Infrared spectra of WTP sludge from two different WTPs.

The Scanning electron microscope (SEM) micrographs of sugarcane bagasse ash, presented in Fig. S7(a) to (f), reveal distinct morphologies associated with different processing conditions. In Fig. S7(a) and (d), a predominantly bulk morphology is observed, with highly agglomerated particles and irregular contours, suggesting ashes with low structural organization. Fig. S7(b) shows a more open structure, with smaller particles dispersed among larger fragments. An increase in microporosity and particle heterogeneity is noted, with interspersed fibrous traces, which may be related to a lower combustion temperature compared to the previous two. In the micrograph of Fig. S7(c), a more preserved fibrous structure is clearly identified, with elongated and porous surfaces. The presence of channels and cavities may indicate a more controlled combustion process that retained part of the original plant fiber morphology. In contrast, Fig. S7(e) stands out due to its lamellar morphology and the presence of elongated, partially collapsed structures, likely remnants of the bagasse's vascular structure. Finally, Fig. S7(f) reveals a highly fibrous and oriented structure, indicating greater preservation of the original biomass morphology. The rough surface and fiber integrity suggest that the sample was subjected to a less severe thermal process, allowing the retention of structural characteristics of the plant-based raw material. This interpretation is supported by previous characterization results, which indicate a high organic matter content in the ash from Mill 3.


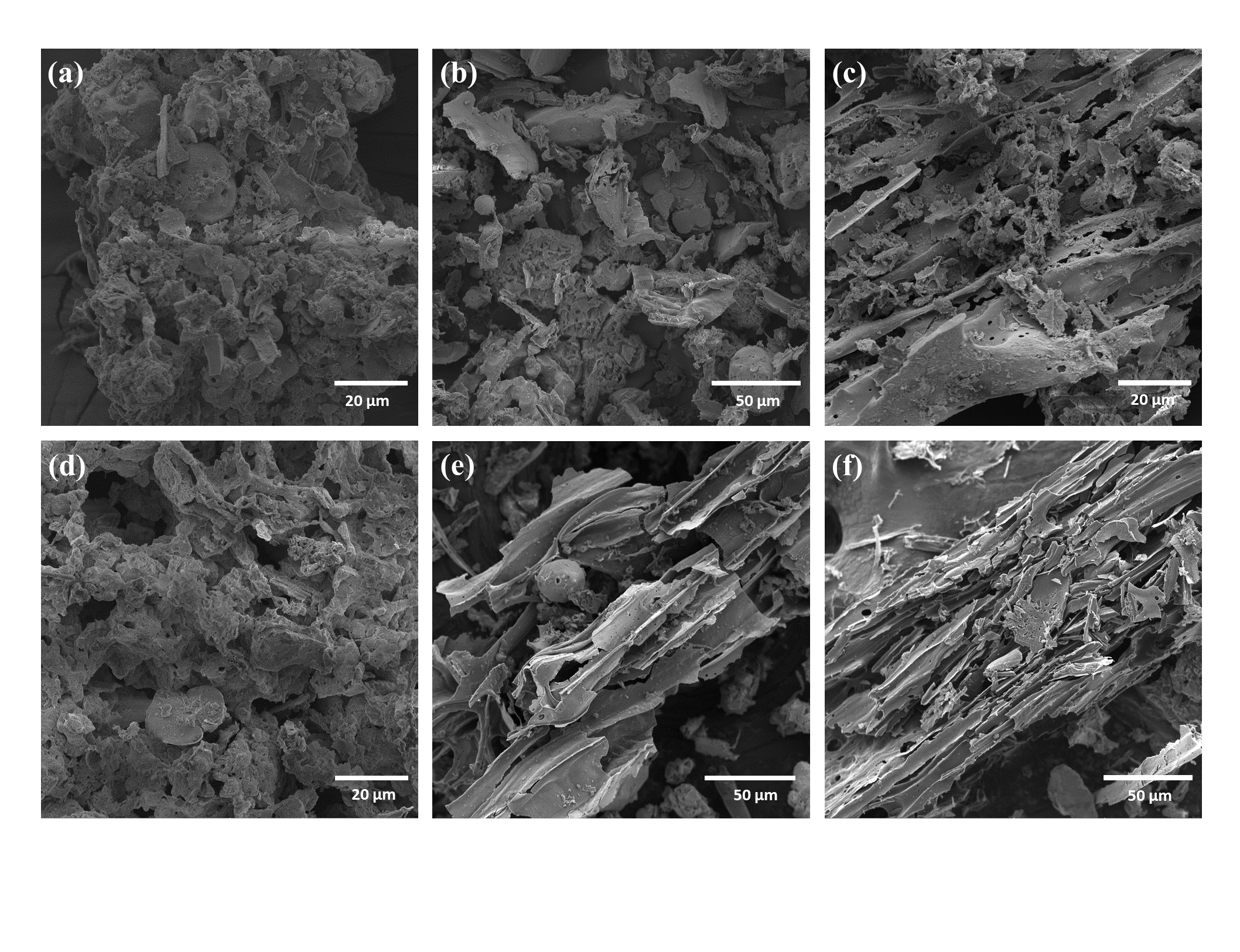


| (a) | Mill 1 – 2019/2020 harvest |
| --- | --- |
| (b) | Mill 1 – 2021/2022 harvest |
| (c) | Mill 1 – 2022/2023 harvest** (This work) |
| (d) | Mill 1 – 2022/2023 harvest* |
| (e) | Mill 2 – 2024/2025 harvest |
| (f) | Mill 3 – 2024/2025 harvest |

**Fig. S7.** SEM images of sugarcane bagasse ash from different sugar mills and harvests (*Ash collected from the upper part of the settling pond; **ash collected from the bottom of the settling pond).

Fig. S8(a) and (b) show micrographs of sludge from two different WTPs, revealing that both exhibit structures composed of agglomerated particles with irregular shapes, which are typical characteristics of this type of waste.


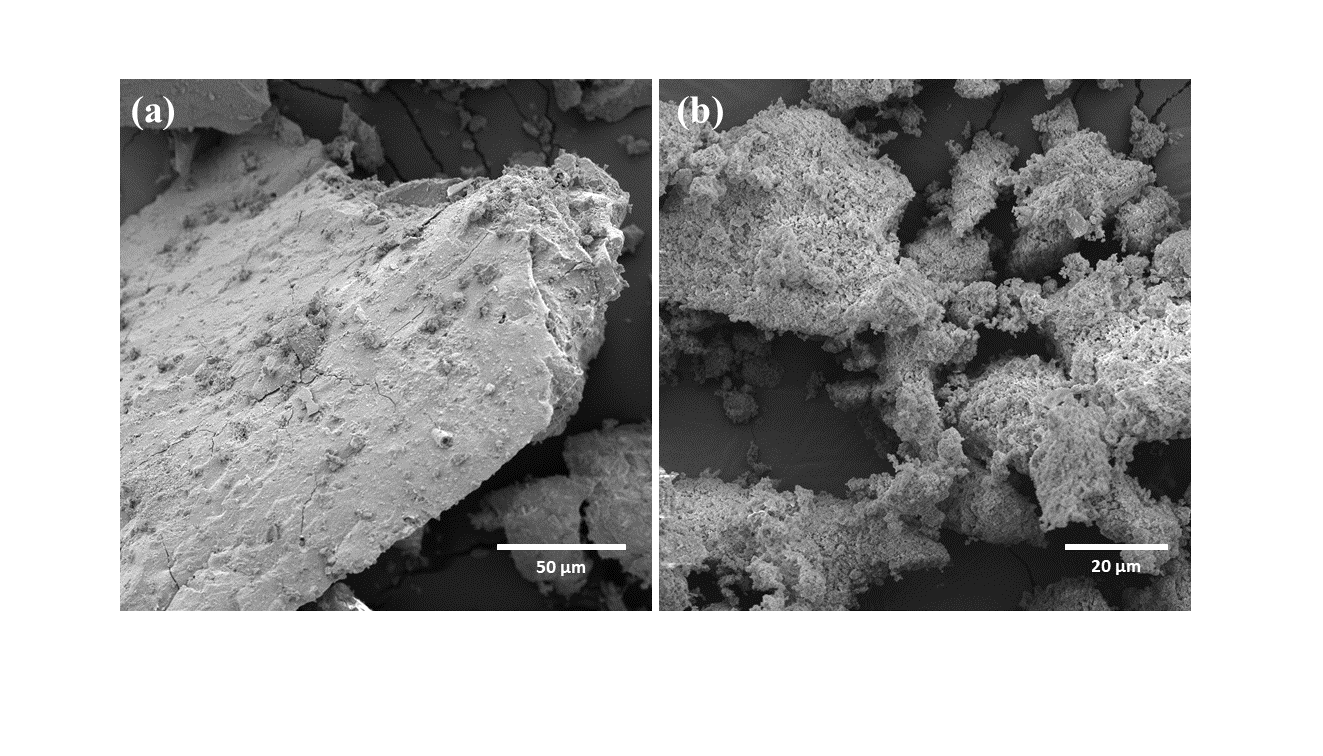


**Fig. S8.** SEM images of sludge samples collected from (a) Agrestina Nova WTP and (b) Gurjaú WTP.

The data obtained indicate that both sugarcane bagasse ash and WTP sludge have potential for applications in zeolite synthesis, especially due to their significant silica and aluminum contents.

**S3. UV-vis spectrum of the dye Acid Red 27**

Figure S9 presents the UV–Vis absorption spectra of Acid Red 27 (AR27) at different pH values (2, 6, and 10). The spectra show that the characteristic absorption bands of AR27 remain essentially unchanged over the evaluated pH range, indicating that the chromophoric structure of the dye is stable under the experimental conditions.





**Fig. S9.** UV–Vis absorption spectra of AR27 recorded at different pH values (pH 2, 6, and 10).

**S4. Kinetic data for additional concentrations (90, 500 and 1000 ppm)**

The kinetic behavior of Acid Red 27 (AR27) adsorption onto the zeolitic compound at higher initial concentrations (90, 500, and 1000 ppm) was also evaluated. The experimental data were fitted to the pseudo-first-order (PFO), pseudo-second-order (PSO), and Intraparticle diffusion (Weber-Morris) models. The corresponding fitting curves are shown in Fig. S10a (PFO and PSO) and Fig. S10b (Weber–Morris), while the estimated kinetic parameters for all models are summarized in Table S5.


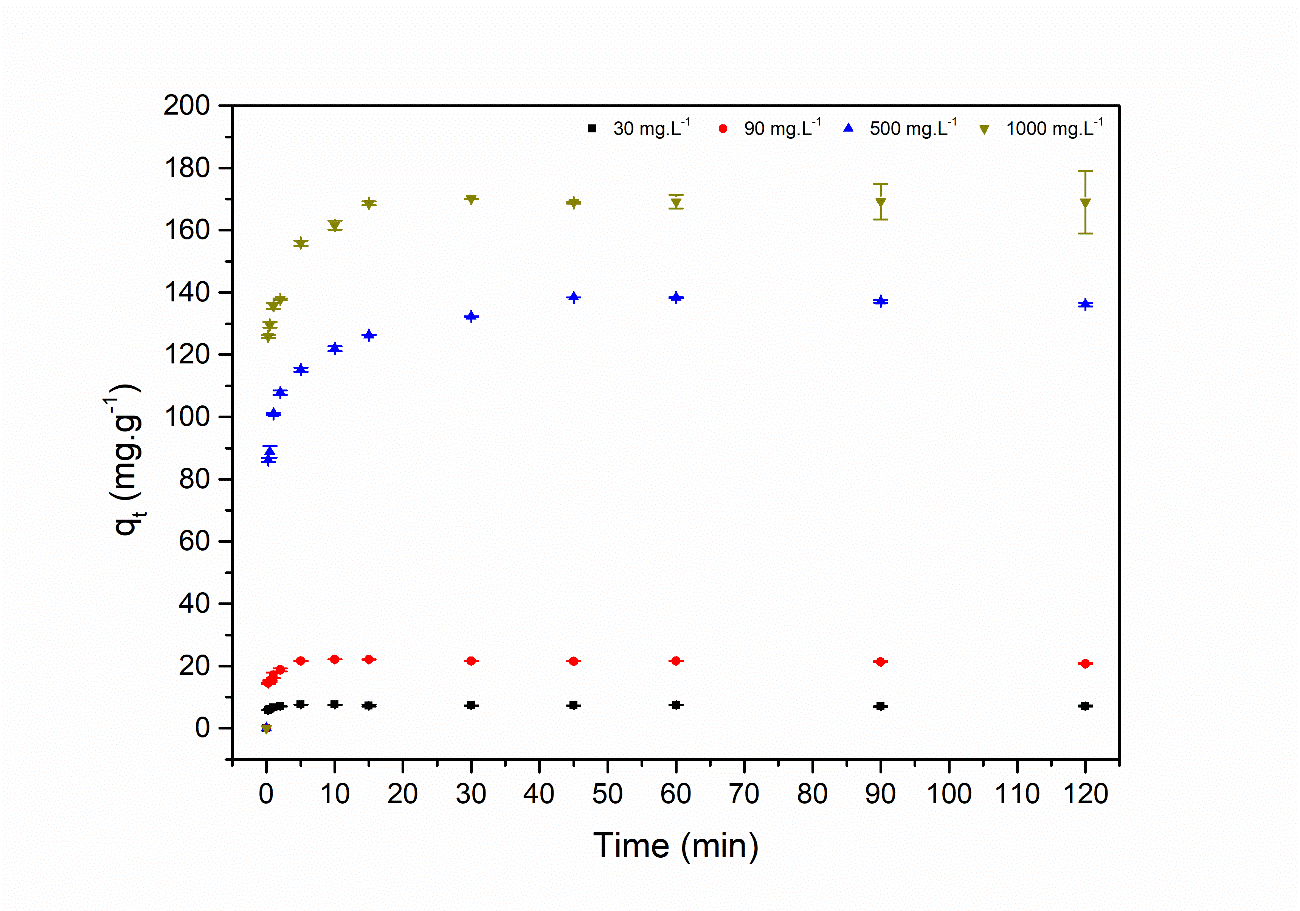


**(a)**


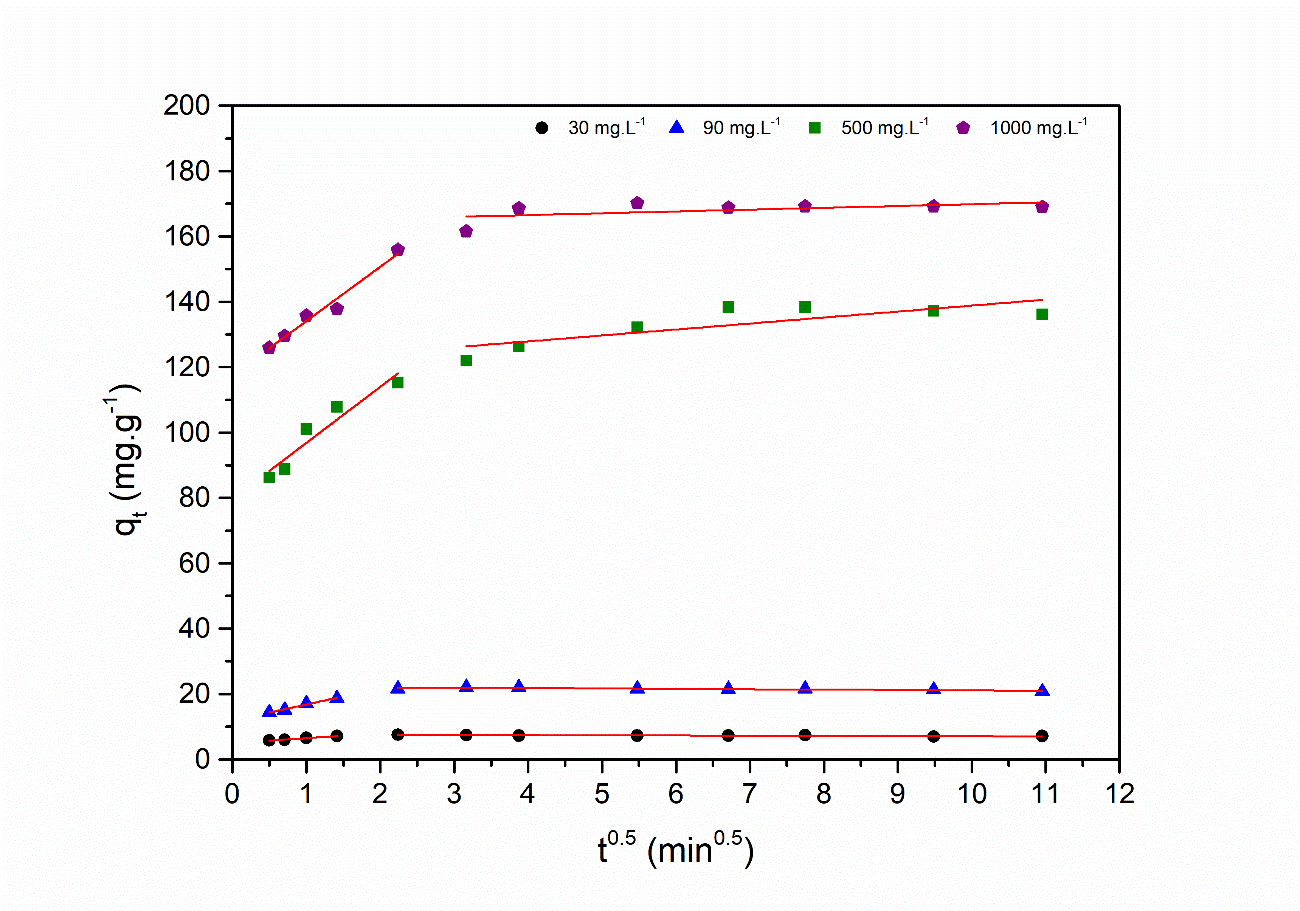


**(b)**

**Fig. S10.** Graphs of kinetic model fits for AR27 adsorption onto the zeolitic compound at several initial concentrations: (a) pseudo-first-order (PFO) and pseudo-second-order (PSO) kinetic models; (b) Weber–Morris intraparticle diffusion model. (Conditions applied: 30, 90, 500, and 1000 mg·L⁻¹ of initial dye concentration at pH₀ 2; 4 g·L⁻¹ of adsorbent; agitation at 300 rpm; temperature fixed at 25 ± 1 °C).

**Table S5**

Kinetic parameters and statistical fit indices (RSS, R², χ²) for AR27 adsorption onto the zeolitic compound at various initial concentrations (Conditions applied: 90, 500, and 1000 mg·L⁻¹ of initial dye concentration at pH₀ 2; 4 g·L⁻¹ of adsorbent; agitation at 300 rpm, temperature fixed at 25 ± 1 °C).

| **Initial concentration** | **Kinetic model** | **Parameter** | **Value** |
| --- | --- | --- | --- |
| 90 mg·L^-1^ | Pseudo-first-order | Predicted q_e_ (mg·g^-1^) | 20.9137 ± 0.5236 |
|  |  | k_1_ (min^-1^) | 3.2975 ± 0.5510 |
|  |  | RSS | 28.6583 |
|  |  | R^2^ | 0.9360 |
|  |  | χ^2^ | 2.6053 |
|  | Pseudo-second-order | Predicted q_e_ (mg·g^-1^) | 21.5036 ± 0.3223 |
|  |  | k_2_ (g·mg^-1^·min^-1^) | 0.2749 ± 0.0413 |
|  |  | RSS | 9.0493 |
|  |  | R^2^ | 0.9797 |
|  |  | χ^2^ | 0.8227 |
|  | Intraparticle diffusion | k_id,1_ (mg·g^-1^·min^-0.5^) | 4.9321 ± 0.3281 |
|  | **(**Weber-Morris model) | C_1_ (mg·g^-1^) | 11.8560 ± 0.3176 |
|  |  | RSS_1_ | 0.1015 |
|  |  | Pearson’s r_1_ | 0.9956 |
|  |  | k_id,2_ (mg·g^-1^·min^-0.5^) | -0.1188 ± 0.0316 |
|  |  | C_2_ (mg·g^-1^) | 22.2846 ± 0.2166 |
|  |  | RSS_2_ | 0.4020 |
|  |  | Pearson’s r_2_ | -0.8375 |
| 500 mg·L^-1^ | Pseudo-first-order | Predicted q_e_ (mg·g^-1^) | 126.2808 ± 4.2157 |
|  |  | k_1_ (min^-1^) | 3.0971 ± 0.67493 |
|  |  | RSS | 1842.0704 |
|  |  | R^2^ | 0.8927 |
|  |  | χ^2^ | 167.4609 |
|  | Pseudo-second-order | Predicted q_e_ (mg·g^-1^) | 130.7194 ± 2.9845 |
|  |  | k_2_ (g·mg^-1^·min^-1^) | 0.0381 ± 0.0081 |
|  |  | RSS | 761.3951 |
|  |  | R^2^ | 0.9556 |
|  |  | χ^2^ | 69.2177 |
|  | Intraparticle diffusion | k_id,1_ (mg·g^-1^·min^-0.5^) | 17.1516 ± 3.0997 |
|  | **(**Weber-Morris model) | C_1_ (mg·g^-1^) | 79.6876 ± 4.1006 |
|  |  | RSS_1_ | 54.4278 |
|  |  | Pearson’s r_1_ | 0.9543 |
|  |  | k_id,2_ (mg·g^-1^·min^-0.5^) | 1.8288 ± 0.6032 |
|  |  | C_2_ (mg·g^-1^) | 120.5562 ± 4.3853 |
|  |  | RSS_2_ | 89.0026 |
|  |  | Pearson’s r_2_ | 0.8048 |
| 1000 mg·L^-1^ | Pseudo-first-order | Predicted q_e_ (mg·g^-1^) | 159.9996 ± 4.2530 |
|  |  | k_1_ (min^-1^) | 4.9677±1,0736 |
|  |  | RSS | 1990.1325 |
|  |  | R^2^ | 0.9223 |
|  |  | χ^2^ | 180.9211 |
|  | Pseudo-second-order | Predicted q_e_ (mg·g^-1^) | 164.7467 ± 3.0353 |
|  |  | k_2_ (g·mg^-1^·min^-1^) | 0.0531 ± 0.0118 |
|  |  | RSS | 831.9773 |
|  |  | R^2^ | 0.9675 |
|  |  | χ^2^ | 75.6343 |
|  | Intraparticle diffusion | k_id,1_ (mg·g^-1^·min^-0.5^) | 16.6233 ± 1.6049 |
|  | **(**Weber-Morris model) | C_1_ (mg·g^-1^) | 117.4844 ± 2.1230 |
|  |  | RSS_1_ | 14.5898 |
|  |  | Pearson’s r_1_ | 0.9863 |
|  |  | k_id,2_ (mg·g^-1^·min^-0.5^) | 0.5589 ± 0.3807 |
|  |  | C_2_ (mg·g^-1^) | 164.2757 ± 2.7678 |
|  |  | RSS_2_ | 35.4560 |
|  |  | Pearson’s r_2_ | 0.5488 |

At all evaluated concentrations, the PSO kinetic model provided the best fit to the experimental data. This was evidenced by consistently higher R² values, indicating a stronger correlation with the observed data, as well as lower RSS and χ² values, reflecting a smaller deviation between predicted and experimental results. These findings suggest that the adsorption process of AR27 dye onto the zeolitic compound is better described by a second-order mechanism, possibly indicating that the adsorption rate is more dependent on the number of active sites occupied than on the dye concentration remaining in solution.

A PSO rate constant (k₂) showed a clear decreasing trend with increasing initial concentration of AR27. This behavior indicates that the adsorption rate is faster at low dye concentrations due to the greater availability of active sites and lower molecular competition. At higher concentrations, the surface of the zeolitic compound approaches saturation, and mass transfer limitations become more pronounced, resulting in slower adsorption kinetics.

**S5. AR27 Molecular Structure and Equilibrium Adsorption Isotherms on Zeolite, GAC, and PAC**


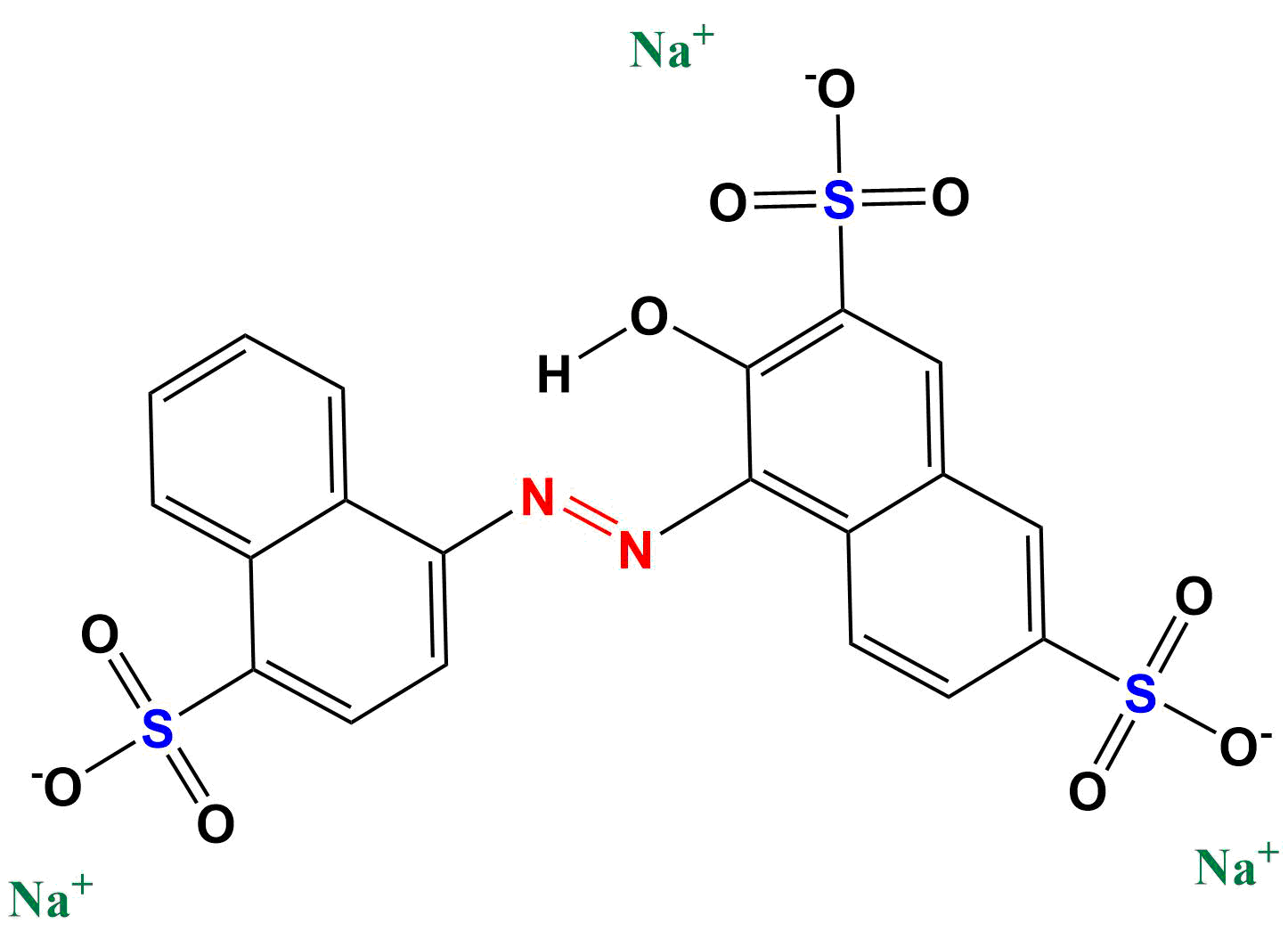


**Fig. S11.** Molecular representation of Acid Red 27 dye structure.


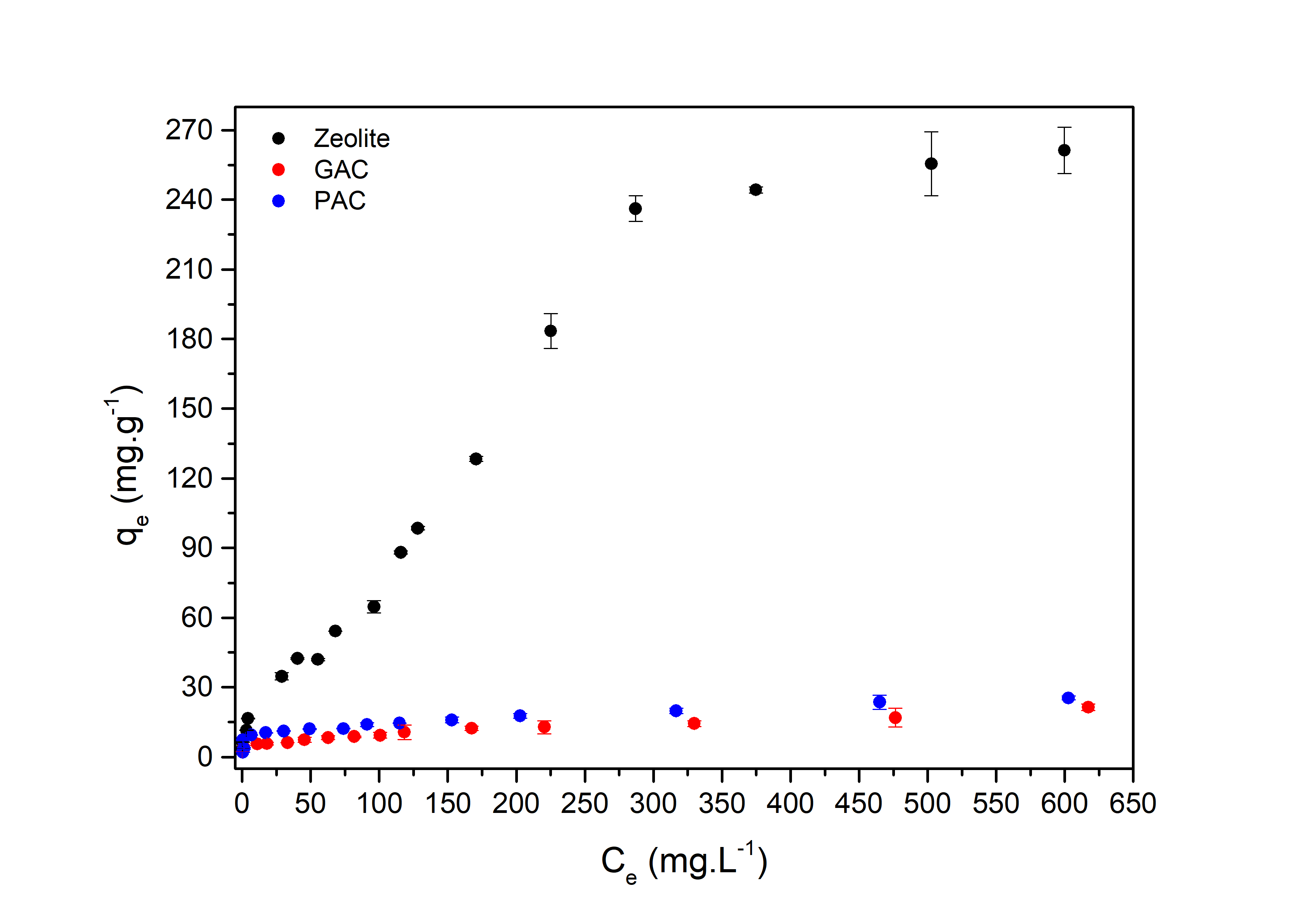


**Fig. S12.** Experimental adsorption equilibrium isotherms of AR27 onto the synthesized zeolite, GAC and PAC. (Conditions applied: initial dye concentrations varying between 5 and 1000 mg·L⁻¹ at pH₀ = 2, adsorbent dosages of 1.5 g·L⁻¹ (zeolite), 2.8 g·L⁻¹ (GAC) and 2.5 g·L⁻¹ (PAC), agitation at 300 rpm, temperature fixed at 25 ± 1 °C, and contact time maintained until equilibrium was achieved)

**REFERENCES**

Bujdák J (2020) Adsorption kinetics models in clay systems. The critical analysis of pseudo-second order mechanism. Applied Clay Science 191:105630. <https://doi.org/10.1016/j.clay.2020.105630>

Davarcioglu B (2011) Spectral characterization of non-clay minerals found in the clays (Central Anatolian-Turkey). International Journal of the Physical Sciences 6:511-522. <https://doi.org/10.5897/JPGE.9000047>

Ebadi A, MohammadZadeh JS S, Khudiev A (2009) What is the correct form of BET isotherm for modeling liquid phase adsorption? Adsorption 15:65-73. <https://doi.org/10.1007/s10450-009-9151-3>

Freundlich H (1907) About adsorption in solutions. Zeitschrift für physikalische Chemie 57:385-470 (in German). <https://doi.org/10.1515/zpch-1907-5723>

Ho Y-S, McKay G (1999) Pseudo-second order model for sorption processes. Process Biochemistry 34:451-465. <https://doi.org/10.1016/S0032-9592(98)00112-5>

Hubbe MA, Azizian S, Douven S (2019) Implications of apparent pseudo-second-order adsorption kinetics onto cellulosic materials: A review. BioResources 14:7582-7626. <https://doi.org/10.15376/biores.14.3.7582-7626>

Langmuir I (1918) The adsorption of gases on plane surfaces of glass, mica and platinum. Journal of the American Chemical Society 40:1361-1403. <https://doi.org/10.1021/ja02242a004>

Ling YP, Tham RH, Lim SM, Fahim M, Ooi CH, Krishnan P, Matsumoto A, Yeoh FY (2017) Evaluation and reutilization of water sludge from fresh water processing plant as a green clay substituent. Applied Clay Science 143:300-306. <https://doi.org/10.1016/j.clay.2017.04.007>

Mali AK, Nanthagopalan P (2020) A systematic assessment for the determination of sugarcane bagasse ash variation potential throughout the harvesting season. Materials Today: Proceedings 32:888-895. <https://doi.org/10.1016/j.matpr.2020.04.511>

Payne JH (2010) Unit operations in cane sugar production. 2nd ed. Nobel; STAB, São Paulo (in Portuguese).

Peres EC, Slaviero JC, Cunha AM, Hosseini–Bandegharaei A, Dotto GL (2018) Microwave synthesis of silica nanoparticles and its application for methylene blue adsorption. Journal of Environmental Chemical Engineering 6:649-659. <https://doi.org/10.1016/j.jece.2017.12.062>

Saikia BJ, Parthasarathy G (2010) Fourier transform infrared spectroscopic characterization of kaolinite from Assam and Meghalaya, Northeastern India. J. Mod. Phys 1:206-210. <https://doi.org/10.4236/jmp.2010.14031>

Şenol ZM, Şimşek S, Ulusoy Hİ, Özer A (2020) Synthesis and characterization of a polyacrylamide-dolomite based new composite material for efficient removal of uranyl ions. Journal of Radioanalytical and Nuclear Chemistry 324:317-330. <https://doi.org/10.1007/s10967-020-07047-2>

Tran HN, Lima EC, Juang RS, Bollinger JC, Chao HP (2021) Thermodynamic parameters of liquid–phase adsorption process calculated from different equilibrium constants related to adsorption isotherms: A comparison study. Journal of Environmental Chemical Engineering 9:106674. <https://doi.org/10.1016/j.jece.2021.106674>
